# Construction of Protein‐Like Helical‐Entangled Structure in Lithium‐Ion Silicon Anode Binders via Helical Recombination and Hofmeister Effect

**DOI:** 10.1002/advs.202412769

**Published:** 2025-04-27

**Authors:** Shiyuan Dai, Fei Huang, Jinglun Yan, Yuan Yuan Sun, Chao Chen, HaiDong Li

**Affiliations:** ^1^ School of Materials Science and Engineering Zhejiang Sci‐Tech University No. 928, No. 2 Street, Xiasha Higher Education Park Hangzhou China; ^2^ Nanotechnology Research Institute Jiaxing University No. 899 Guangqiong Road Jiaxing China; ^3^ College of Chemistry and Chemical Engineering Yangzhou University 88 South Daxue Road Yangzhou China; ^4^ G60 STI Valley Industry & Innovation Institute Jiaxing University Building No. 7, Jiaxing Intelligence and Innovation Park Jiaxing China; ^5^ Shanghai Institute of Applied Physics Chinese Academy of Science No. 2019 Jialuo Road, Jiading District Shanghai China

**Keywords:** binder, helical‐entangled network, Hofmeister effect, Lithium‐ion battery, Silicon anode, Thermo‐Responsive

## Abstract

In this study, a novel gelatin‐xanthan gum composite binder is successfully developed with a protein‐like helical‐entangled network structure through thermo‐responsive and Hofmeister effect to improve the cycling stability of silicon anodes in lithium‐ion batteries. As the temperature changes, the molecular chains of xanthan gum and gelatin undergo de‐helixing, intertwining, and co‐helixing, ultimately self‐assembling into a protein‐like spatial structure. Furthermore, immersing in Hofmeister salt solution enhances the degree of helical entanglement, significantly improving strength and toughness. This novel helical‐entangled structure absorbs and dissipates the stress and strain caused by silicon volume expansion through repeated bending, twisting, and stretching, similar to protein spatial structures, thereby maintaining the integrity of the silicon anode and enhancing its cycling stability. The silicon anode with the optimized binder exhibits high initial Coulombic efficiency, favorable rate performance, and long‐term cycling stability. At a current density of 0.5 A g⁻¹, the silicon anode has a specific capacity of 1779.8 mAh g⁻¹ after 300 cycles, with a capacity retention rate of 80.65%. This study demonstrates the feasibility of natural polymers forming complex 3D network structures through self‐assembly and intermolecular forces, providing a new approach for the design of silicon anode binders.

## Graphical abstract

This study presents a gelatin‐xanthan gum composite binder featuring a protein‐like helical‐entangled structure, formed through thermo‐responsive recombination and the Hofmeister effect. This innovative structure, akin to protein behavior, absorbs stress and strain, thereby enhancing the cycling stability and rate performance of silicon anodes in lithium‐ion batteries.

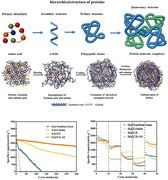

## 1 Introduction

Recent advancements in renewable energy and industrial automation have expanded the application of lithium‐ion batteries from electronic devices to electric vehicle energy storage.^[^ ^1^ ^]^ However, the theoretical specific capacity of graphite anodes is 372 mAh g⁻¹, which falls short of high‐capacity requirements. Silicon, with a theoretical specific capacity of 4200 mAh g⁻¹, presents an ideal alternative for LIBs.^[^ ^2^ ^]^ Nevertheless, silicon anodes undergo volumetric expansion of up to 300% during lithiation and delithiation, leading to fragmentation, unstable solid electrolyte interphase (SEI), and electrode structure degradation, thereby reducing cycling stability.^[^ ^3^ ^]^ To address these challenges, researchers have explored strategies such as electrolyte additives,^[^ ^4^ ^]^ Si nanostructure (SiNPs)  modification,^[^ ^5^ ^]^ silicon‐based composites, and the development of novel binders^[^ ^1^, ^6^ ^]^ to enhance the cycling stability of silicon anodes. Among these approaches, designing a high‐performance binder compatible with silicon, to replace traditional binders like Polyvinylidene Fluoride(PVDF) that lack polar functional groups and exhibit weak interactions with SiNPs,^[^ ^7^ ^]^ is a relatively simple and effective method with promising industrial application prospects.

Among the reported binders, a series of natural polymer binders such as sodium alginate (Alg),^[^ ^8^ ^]^ chitosan (CS),^[^ ^9^ ^]^ xanthan gum,^[^ ^10^ ^]^ guar gum,^[^ ^11^ ^]^ carrageenan,^[^ ^12^ ^]^ and locust bean gum (LBG)^[^ ^13^ ^]^ have attracted considerable attention from the research and industrial communities. The polar functional groups of these natural polymers form reversible hydrogen bonds with SiNPs, significantly enhancing the interfacial adhesion with silicon.^[^ ^14^ ^]^ Furthermore, these natural polymers are capable of constructing three dimensional (3D) network structures through chemical or physical crosslinking, thereby improving the stability of the electrode structure. The synergistic effect of these two factors can, to some extent, inhibit and buffer the severe stress changes caused by the volume expansion of silicon, thereby improving the cycling stability of silicon anodes. However, such 3D network structures often lack sufficient mechanical strength and toughness to effectively absorb and dissipate the stresses and strains caused by the volumetric changes of silicon over the long term, ultimately leading to a significant reduction in specific capacity after prolonged cycling. To address this issue simply and effectively, we turn our attention to the helical structures of natural polymer chains. During the prolonged evolutionary process of nature, the molecular chains of plants and animals frequently exhibit a helical structure. These helices assemble to form more complex hierarchical structures known as protein complexes. This intricate, layered helical‐entangled structure can absorb and dissipate stress through natural bending, twisting, and stretching^[^ ^15^ ^]^ thereby reducing destructive strain and meeting the survival needs of plants and animals. Inspired by this, we attempt to recombine different molecular chains into helical structures similar to proteins, aiming to better utilize the helical secondary structure of natural polymer chains to improve the strength and toughness of binders.

Among natural polymers with helical molecular structures, xanthan gum stands out as an acidic polysaccharide produced by the fermentation of Xanthomonas bacteria. Its rigid double‐helical structure is stabilized by hydrogen bonding.^[^ ^16^ ^]^ This structure makes xanthan gum a potential framework material for 3D networks.^[^ ^17^ ^]^ Gelatin, derived from the hydrolysis of collagen, forms a triple‐helical structure through the interweaving of peptide chains with amino and carboxyl groups.^[^ ^18^ ^]^ When xanthan gum and gelatin are combined, the amino‐rich residues of gelatin attach to the xanthan gum molecular chain via electrostatic and hydrogen bonding, thus providing a large number of hydrogen‐bonding sites and bridging the xanthan gum molecular chains.^[^ ^19^ ^]^ Due to the thermo‐responsive nature of the helical structures, the molecular chains of xanthan gum and gelatin undergo a process of de‐helixing, mutual entanglement, and co‐helixing during heating and then the cooling process in solution, ultimately self‐assembling into a multi‐level spatial structure similar to proteins. In this protein‐like helical‐entangled network, the entangled region provides a stable framework structure for the whole network, while the helical regions buffer the stress and strain caused by the volumetric expansion of silicon. Furthermore, we utilize the salting‐out effect in the Hofmeister sequence to promote the degree of helical‐entangled of xanthan gum‐gelatin by immersion in kosmotropic salt solution, which further enhances the strength and toughness of the novel binder.

Ultimately, this study constructed a binder material with a helical‐entangled network structure through the helical recombination between the double‐helix long chains of xanthan gum and the triple‐helix branched short chains of gelatin. By adjusting the ratio of xanthan gum to gelatin and utilizing the Hofmeister salting‐out effect, the helical‐entangled network of xanthan gum and gelatin was optimized, resulting in superior performance. GX was characterized by various methods, including Fourier Transform Infrared Spectroscopy (FTIR), X‐ray Diffraction (XRD), compression modulus testing, and 180° peel testing. The results indicated that as the degree of helical entanglement and cross‐linking within the polymer increased, the structure of the helical‐entangled network became more stable, thereby enhancing the strength and toughness of the binder material. Additionally, the electrochemical performance of silicon anodes prepared with different binders was investigated through a series of constant current charge–discharge tests and Electrochemical Impedance Spectroscopy (EIS) measurements. The test results demonstrated that the prepared helical‐entangled network binder material could effectively suppress and buffer the volume expansion of silicon, maintain the structural integrity of the silicon‐based anode, and thereby improve the cycling performance of lithium‐ion batteries. This study achieved more efficient utilization of natural polymer binder materials through the regulation of molecular chain conformation, providing an innovative approach for the preparation of novel binder materials.

## 2 Results and Discussion

**Figure 1:**
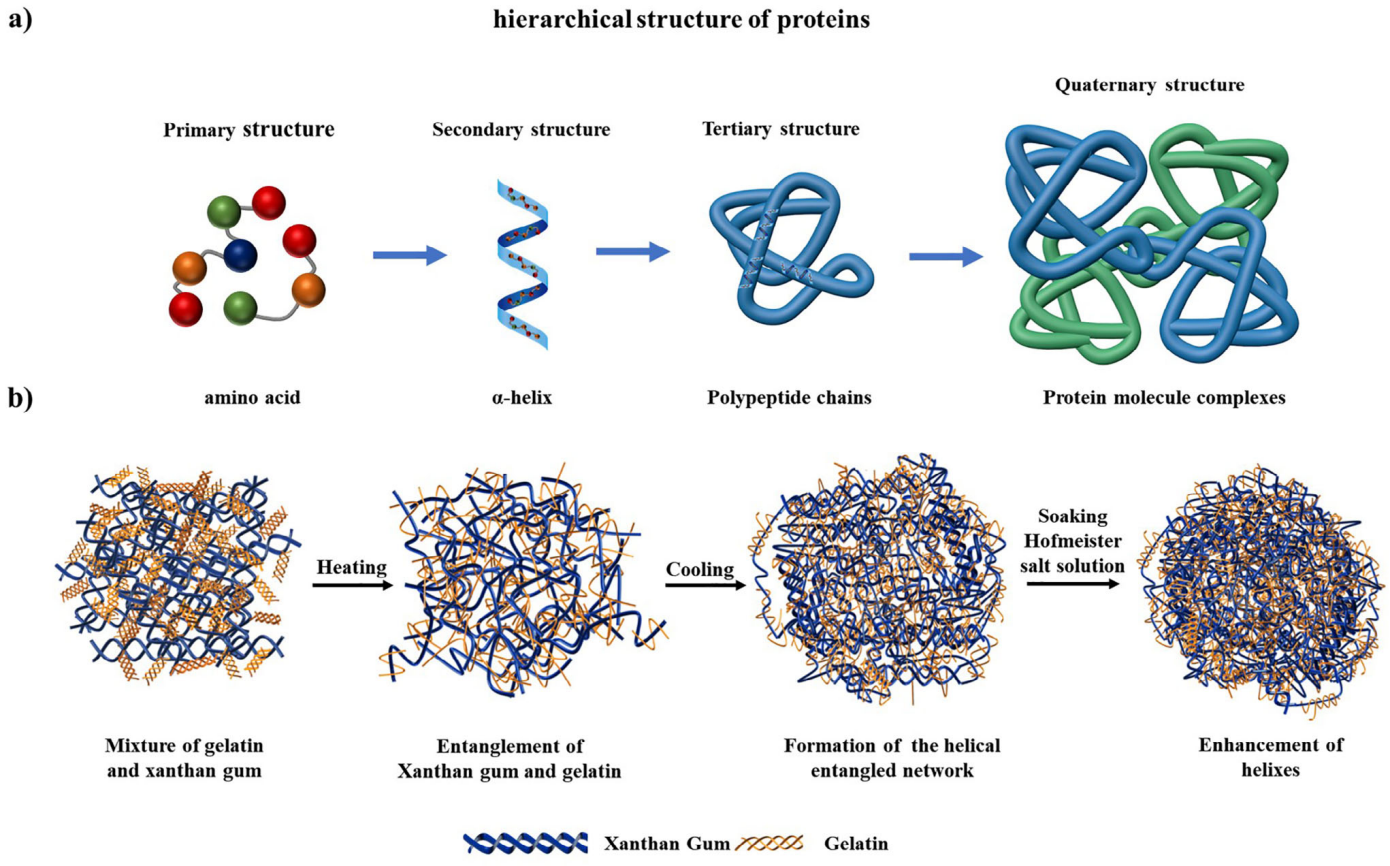
a) The hierarchical structure of proteins; b) The recombination and enhancement of the helical‐entangled network.

**Figure** **1** illustrates the assembly and enhancement mechanism of the helical‐entangled network structure formed by gelatin and xanthan gum. The recombination process is akin to the self‐assembly of peptide chains into protein complexes. Additionally, we leverage the salting‐out effect within the Hofmeister series by immersing the gels in a kosmotropic salt solution, which enhances the helical entanglement and subsequently fortifies the mechanical properties of the network.

Initially, in a high‐temperature solution, the helical molecular chains of gelatin and xanthan gum unravel and extend into coil‐like structures, attracting each other through electrostatic and hydrogen bonding interactions. During this process, electrostatic interactions dominate, shortening the distance between molecular chains and promoting aggregation. Hydrogen bonds enhance and synergize this process, helping to form and stabilize aggregates.^[^ ^19^, ^20^ ^]^ During the formation of xanthan gum and gelatin aggregates, the primary driving force for polymer mixing is weak intermolecular forces, with mixing entropy playing a dominant role. This promotes the entanglement of molecular chains, allowing xanthan gum and gelatin to spontaneously form an entangled network structure.^[^ ^21^ ^]^ This process is analogous to the formation of stable helices and entangled secondary structures in proteins, where multiple peptide chains interact through hydrogen bonding. Upon cooling, the interaction between xanthan gum and gelatin diminished, leading to a reduction in charge density and a decrease in intermolecular distances. Both gelatin and xanthan gum underwent a coil‐to‐helix transition, resulting in the formation of a novel helical‐entangled network structure. This process is analogous to the folding of protein secondary structures into a 3D tertiary network. The formation of a protein‐like tertiary structure between xanthan gum and gelatin demonstrates the potential of natural polymers to self‐assemble into complex 3D network structures through intermolecular forces.

To validate the helical recombination process between xanthan gum and gelatin, xanthan gum, gelatin, and GX were tested by viscosity, rheometer analysis, differential scanning calorimetry (DSC), and FTIR tests.

**Figure 2:**
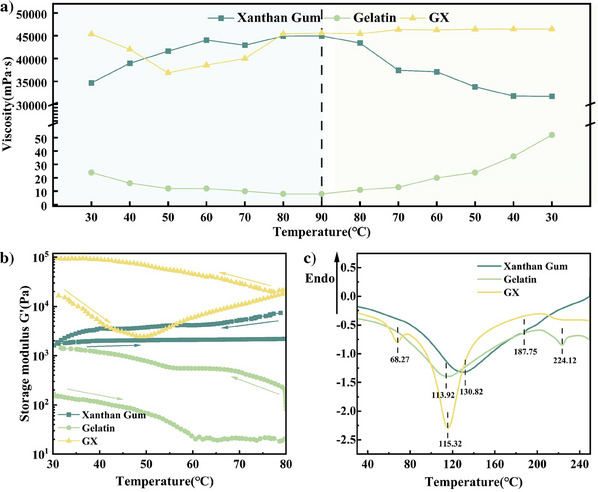
a) Temperature‐viscosity curves of xanthan gum, gelatin, and GX; b) Evolution of *G'* during heating‐cooling of the three systems, with heating rate = 5 °C min^−1^ and scanning rate of 1 rad/s; c) DSC heating curves. Scan rate = 1 °C min^−1^.

As illustrated in **Figure** **2**a, the viscosity of gelatin, xanthan gum, and GX exhibits distinct trends with temperature. During heating, gelatin transitioned from a triple helix structure to a random coil structure. This “helix‐to‐coil” transition resulted in a decrease in viscosity with increasing temperature, primarily due to the short molecular chains of gelatin, which made it difficult to form effective entanglements between the chains. In contrast, the viscosity curve of xanthan gum was opposite to that of gelatin, increasing with increasing temperature until stabilizing at 80 °C. The primary reason was that as xanthan gum molecular chains transitioned from helix to coil, the fully extended long chains became entangled, leading to an increase in solution viscosity.^[^ ^22^ ^]^ For GX, the viscosity was significantly higher than that of either xanthan gum or gelatin solutions alone at the initial heating stage, indicating that gelatin had already attached to the xanthan gum molecular chains at room temperature through electrostatic and hydrogen bonding interactions, acting as a bridge for xanthan gum. As the temperature increased, the viscosity of GX first decreased and then increased, mirroring the temperature‐dependent behaviors of gelatin and xanthan gum, respectively. When the temperature reached 80 °C, the viscosity stabilized, indicating that the molecular chains of xanthan gum and gelatin had fully unwound from their helices and entangled with each other. During the cooling process, since xanthan and gelatin were already fully entangled, their molecular chains began to reform helices together, eventually forming a helical‐entangled network structure similar to the spatial structure of proteins. The spatial structure of proteins is typically stabilized by hydrogen bonds, hydrophobic interactions, and electrostatic interactions.

To further investigate the rheological properties of xanthan gum, gelatin, and GX at different temperatures, we conducted temperature‐dependent rheological measurements using a rotational rheometer to determine the storage modulus of the hydrogels (as shown in Figure 2b). The results revealed that the storage modulus of xanthan gum, gelatin, and GX exhibited a similar trend as the viscosity test results. Specifically, the storage modulus of xanthan gum slightly increased with rising temperatures and then decreased during cooling. At higher temperatures, the molecular chain segments of xanthan gum became more mobile, leading to more physical entanglements between the molecular chains, thereby increasing the storage modulus. However, as physical entanglements gradually decreased during cooling, the storage modulus decreased.^[^ ^23^ ^]^ For gelatin, the storage modulus significantly decreased with increasing temperature because the hydrogen bonds in the gelatin molecular chains were broken, and the molecular chain conformation transferred from helical structure to coil structure. However, when the temperature returned to 30 °C, the gelatin molecular chains reformed hydrogen bonds and returned to the gel state, resulting in an increase in storage modulus.^[^ ^24^ ^]^ The storage modulus of the GX mixture decreased initially during heating and then increased, with the storage modulus also slowly increasing during cooling. This phenomenon may be due to the unentanglement and subsequent re‐entanglement of gelatin and xanthan gum molecular chains in the GX mixture during heating. Initially, the molecular chains gradually unwound from the helical‐entangled structure, leading to a decrease in storage modulus. However, as the temperature continued to rise in the later stages of heating, the molecular chains re‐entangled to form new helical structures, causing the storage modulus to increase. During cooling, the molecular chain conformation gradually stabilized, forming a more tightly entangled network structure, and the storage modulus slowly increased.^[^ ^25^ ^]^ Furthermore, the storage modulus of GX is always higher than that of pure xanthan gum and gelatin. This indicates that the GX mixture has significant advantages in molecular chain conformation and physical entanglement, resulting in superior elastic performance.^[^ ^26^ ^]^ The storage modulus is an important parameter reflecting the material's elasticity, and the higher the value, the better the material can recover to its original state after deformation.^[^ ^27^ ^]^ Therefore, it can be seen that the GX mixture has significant advantages in elastic performance and can better maintain the shape and structure of the silicon anode. From the perspective of the slurry casting process, increasing the temperature of the slurry allowed the molecular chains to unfold and form a 3D network structure, which facilitated the adhesion of SiNPs and conductive agents to the electrode foil.^[^ ^28^ ^]^ The subsequent cooling process caused the entangled molecular chains in the slurry to co‐helix, thereby more tightly encapsulating the SiNPs and conductive agents within the network.

The DSC analysis results (Figure 2c) indicated that the pure xanthan gum sample exhibited endothermic peaks at 130.82 and 187.75 °C, while the pure gelatin sample showed endothermic peaks at 113.92 and 224.12 °C. These peaks corresponded to the glass transition temperature and the melting process, respectively. In contrast, the DSC curve of the GX mixture shows peaks at 68.27 and 115.32 °C, which may be due to the dense polymer network structure lowering the freezing point of the hydrogel.^[^ ^29^ ^]^ Furthermore, the incorporation of gelatin acted as a plasticizer for xanthan gum at the molecular level,^[^ ^30^ ^]^ reducing the glass transition temperature of the mixture and enhancing the mobility of the molecular chain segments.^[^ ^31^ ^]^

**Figure 3:**
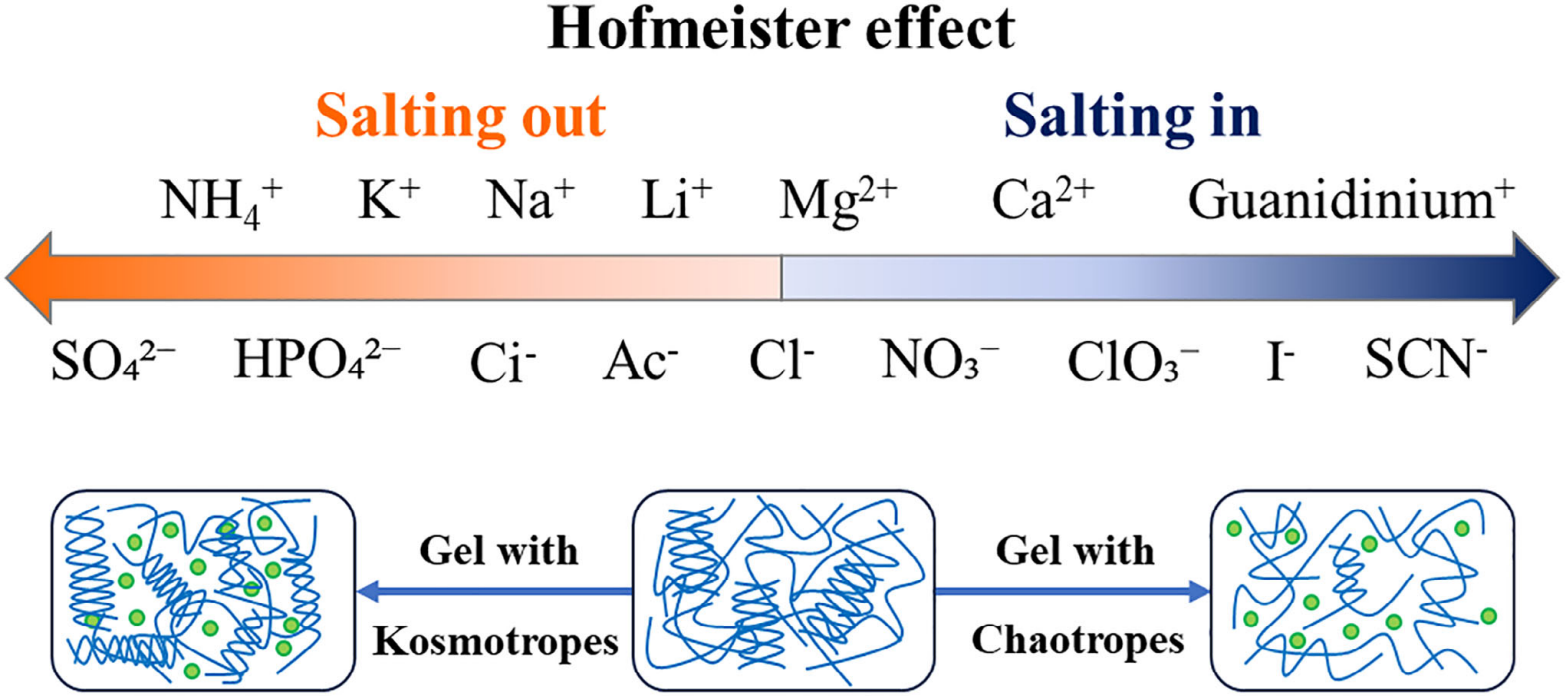
Simplified sequence of the Hofmeister effect and conformational changes of gelatin and xanthan gum in Hofmeister salt solution.

To further enhance the degree of helical entanglement in the network, the prepared silicon anode copper foil was immersed in a Hofmeister salt solution. As a phenomenon widely observed in the biochemical field, the Hofmeister series reveals the different effects of various salt ions on the stability, solubility, and denaturation of colloidal solutions, which depend on the specific interactions between the ions, water, and colloidal molecules (**Figure** **3**).^[^ ^32^ ^]^ In accordance with the influence of salt ions on the solubility of colloidal particles, the Hofmeister series classifies salt ions into two distinct categories: kosmotropic ions and chaotropic ions. Kosmotropic ions are located to the left of chloride ions and enhance the hydration layer and neutralize charges, promoting the orderly arrangement of hydrogen bonds in colloidal particles, leading to precipitation (salting‐out). In contrast, chaotropic ions are located to the right of chloride ions and increase charge density, enhancing electrostatic repulsion and weakening hydrogen bonding in colloidal particles, leading to dissolution (salting‐in).^[^ ^33^ ^]^ Therefore, the mechanical properties of hydrogels can be regulated by immersing them in different types of Hofmeister salt solutions.^[^ ^34^ ^]^ Specifically, in a kosmotropic salt solution (ammonium sulfate, AS), the ammonium ions (NH₄⁺) and sulfate ions (SO₄^2^⁻) can induce stronger interactions between or within polymer chains, promoting polymer aggregation and thereby enhancing the strength and stability of the helical‐entangled network.

**Figure 4:**
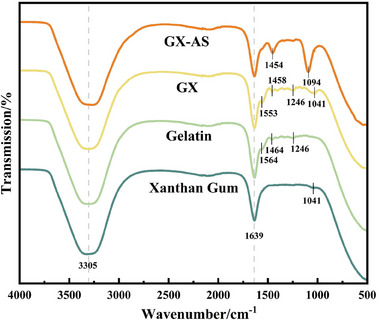
FTIR spectra of xanthan gum, gelatin, GX, and GX‐AS.

To evaluate the interactions between xanthan gum and gelatin and their molecular conformational changes, FTIR analysis was conducted. As shown in **Figure** **4**, Xanthan gum exhibited characteristic peaks at 3305, 1639, and 1041 cm⁻¹, corresponding to the stretching vibrations of OH groups, carboxyl groups, and the C─O─C of glucose units, respectively.^[^ ^35^ ^]^ Gelatin showed characteristic peaks at 3305, 1639, 1564, and 1246 cm⁻¹, corresponding to NH groups, amide I band, amide II band, and amide III band, respectively.^[^ ^36^ ^]^ Additionally, the peak at 1464 cm⁻¹ corresponded to the bending vibration of the methylene groups.^[^ ^37^ ^]^ When xanthan gum and gelatin were mixed, the characteristic peaks of xanthan gum and gelatin overlapped ≈3305 and 1639 cm⁻¹, while the peaks at 1553 and 1246 cm⁻¹ were attributed to gelatin, and the peak at 1041 cm⁻¹ was attributed to xanthan gum. Compared to the individual gelatin and xanthan gum, these peaks in the mixture shifted to lower wavenumbers, primarily due to the formation of hydrogen bonds and ionic bonds between them.^[^ ^19^, ^38^ ^]^ After the mixture was immersed in AS solution, the peaks at 1454 and 1094 cm⁻¹ significantly intensified, mainly due to the characteristic peaks of sulfate ions from the residual AS.^[^ ^34a^ ^]^

In order to investigate the impact of the Hofmeister series on the secondary structure of the materials, the deconvolution method was applied to the FTIR data of the amide I band (1600–1700 cm⁻¹) to analyze the conformational changes of gelatin, xanthan gum, and GX before and after immersion (Figure S1, Supporting Information). The wavenumber assignments within the 1600–1700 cm⁻¹ range are as follows: α‐helix structures correspond to the 1646–1660 cm⁻¹ range, β‐sheet structures to the 1600–1637 cm⁻¹ and 1681–1698 cm⁻¹ ranges, with peaks in the 1638–1645 cm⁻¹ range representing random coils, and β‐turns located between 1661 and 1680 cm⁻¹.^[^ ^39^ ^]^ **Table** **1** shows the content of α‐helix, β‐sheet, β‐turn, and random coil in the secondary structures of xanthan gum, gelatin, GX, and GX‐AS. Compared to GX, the secondary structure of GX‐AS shows a decrease in the content of β‐sheet, β‐turn, and random coil, while the content of α‐helix increases. This indicates that ammonium sulfate has a stabilizing effect on GX, enhancing its α‐helix content, thereby improving its elasticity and strength. This may be due to the strong attraction of the cation to water molecules, forming a more stable hydration shell, which increases the interaction between xanthan gum and gelatin, reduces the entropy of GX molecules, and promotes the formation of α‐helix.^[^ ^40^ ^]^

**Table 1:** Attribution and area percentages of subpeaks of xanthan gum, gelatin, GX, and GX‐AS IR spectra.

Amide I Band Peak Attribution	Amide I. Peak Area Ratio [%]			
	Xanthan Gum	Gelatin	GX	GX‐AS
α‐helix	16.32	20.61	17.74	30.45
β‐sheet	44.32	33.35	41.27	36.82
random coil	19.29	22.50	20.34	16.13
β‐turn	20.07	23.54	20.65	16.60

**Figure 5:**
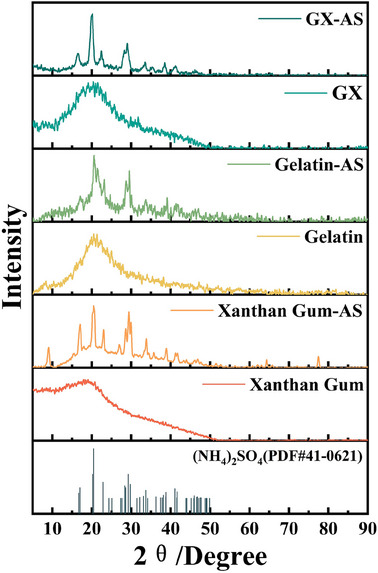
XRD spectra of xanthan gum, gelatin, and GX before and after immersion in ammonium sulfate solution.

XRD was also used to analyze the crystallinity and secondary structure changes of Xanthan Gum, gelatin, and GX before and after immersion in AS solution. As shown in **Figure** **5**, before immersion, the XRD patterns of xanthan gum, gelatin, and GX exhibited a broad peak ≈20.9°, corresponding mainly to the helical structure diffraction peaks of their molecular chains.^[^ ^41^ ^]^ After treatment with AS, the characteristic diffraction peaks of xanthan gum, gelatin, and GX samples became sharper, which may be due to the salting‐out effect of the Hofmeister series promoting the formation of xanthan gum double helices and gelatin triple helices,^[^ ^42^ ^]^ consistent with FTIR analysis results. Additionally, new peaks were observed at 16.9°, 20.4°, and 29.3° in the XRD spectra of Xanthan Gum‐AS, Gelatin‐AS, and GX‐AS samples, respectively, which might be attributed to residual AS salts from the immersion process.

Furthermore, scanning electron microscope (SEM) images revealed that the freeze‐dried gelatin and xanthan gum predominantly exhibited a lamellar morphology (Figure S2a,b, Supporting Information). In contrast, the GX sample demonstrated a uniformly porous structure (Figure S2c,d, Supporting Information), primarily attributed to the helical‐entanglement of xanthan gum and gelatin molecular chains, forming a protein‐like spatial configuration. During the freeze‐drying process, as ice crystals sublimated, hydrogen bonding between the xanthan gum and gelatin molecular chains within this spatial configuration resulted in the formation of a porous network structure.^[^ ^43^ ^]^ After immersion in AS solution, the GX‐AS sample exhibited more densely packed pores, likely due to the Hofmeister salting‐out effect enhancing the extent of helical‐entanglement, thereby creating a more compact network structure.

**Figure 6:**
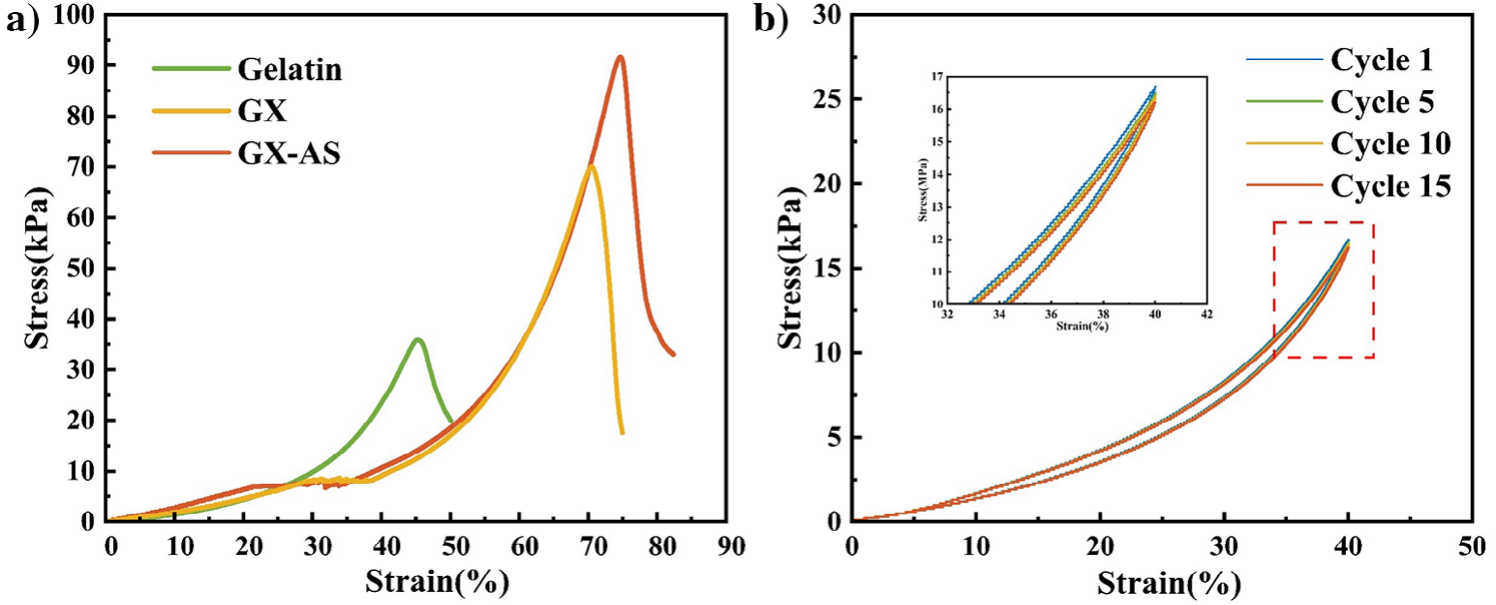
Mechanical property test curves: a) compressive stress‐strain curve; b) fatigue resistance curve of GX‐AS under loading‐unloading cyclic test.

To investigate the interactions between xanthan gum and gelatin, as well as the impact of the Hofmeister effect on the mechanical properties of the system, compression modulus tests were conducted on gelatin, GX, and GX‐AS. It should be noted that xanthan gum, being a non‐gelling polysaccharide in solution, cannot form a gel and thus cannot be subjected to compression modulus testing.^[^ ^44^ ^]^ As shown in **Figure** **6**a, the compression strain and compressive Young's modulus of gelatin hydrogel were only 45.27% and 35.82 kPa, respectively. This was likely due to the shorter molecular chains of gelatin, which were unable to effectively resist external forces, leading to deformation and rupture of the hydrogel.^[^ ^45^ ^]^ When the mass ratio of gelatin to xanthan gum was optimized, the compression strain and modulus of GX hydrogel increased to 70.62% and 70.28 kPa, respectively (Figure S3, Supporting Information). The excellent mechanical properties of the optimized GX hydrogel were likely related to its protein‐like helical‐entangled network structure. To further enhance the mechanical properties of the hydrogel, GX hydrogels were immersed in ammonium salt solutions with different anions, and compression modulus tests were conducted on the immersed hydrogels (Figure S4, Supporting Information). The results indicated that the enhancement in the mechanical properties of the immersed GX hydrogels followed the ion sequence of the Hofmeister series, with AS showing the most significant effect. The compression strain and modulus of GX‐AS immersed in AS increased to 74.84% and 91.40 kPa, respectively, mainly due to the salting‐out effect of the Hofmeister series, which further strengthened the helical‐entangled of the molecular chains.

To further characterize the effects of salt immersion on the fatigue resistance and strain recovery properties of the hydrogel, loading‐unloading compression cycle tests were conducted on GX‐AS (Figure 6b). When the compression strain was set to 40%, the loading‐unloading curves of GX‐AS after fifteen cycles nearly overlapped compared to GX before immersion (Figure S5, Supporting Information). This phenomenon indicated that GX‐AS hydrogel effectively returned to its original shape after repeated compression and release, demonstrating excellent elasticity and recovery ability. The overlapping curves reflected low energy dissipation during the cycles, high internal structural stability, and superior fatigue resistance.^[^ ^46^ ^]^ This was likely due to the salt immersion treatment promoting the formation of hydrogen bonds within the hydrogel, increasing the helical entanglement of the molecular chains, and forming a tighter helical‐entangled network structure. Consequently, the hydrogel efficiently absorbed and released energy during loading and unloading without causing permanent deformation.

Additionally, the mechanical performance test on dried films of gelatin, xanthan gum, GX, and GX‐AS was also conducted. The tensile test results of the dried film samples (Figure S6, Supporting Information) exhibited similar trends to the compression test of the gels.

In summary, the mechanical properties of GX are significantly enhanced, primarily due to the xanthan gum and gelatin molecular chains undergoing de‐helix, intertwining, and co‐helix processes, forming a multi‐level spatial structure similar to proteins. When subjected to external forces, GX can effectively dissipate stress and strain, similar to the helical structure of proteins. The rigid molecular chains of xanthan gum act as the “framework” of the network, providing the initial force transmission path. Through sliding, deformation, and rearrangement of the chain segments, the force is transmitted to multiple helical molecular chains. The stretching deformation of the helices results in the breaking of intramolecular hydrogen bonds, consuming energy and effectively buffering and dissipating the stress and strain caused by the expansion of the silicon volume. To better understand and verify the structural evolution of the xanthan gum‐gelatin network with temperature changes, as well as its structural changes under external forces, we conducted Molecular Dynamics (MD) simulations (as shown in Figure S7, Supporting Information). The simulation results aligned with the proposed mechanism. Furthermore, by soaking in Hofmeister salt solutions, the mechanical properties of GX were significantly enhanced, and the stronger the salting‐out effect, the greater the improvement in mechanical properties. This may be due to the kosmotropic salt ions enhancing the helical entanglement of the network, making it more compact, thereby allowing the helical regions within the network to more effectively dissipate external stress and strain like a spring, maintaining its structural stability.^[^ ^34a^ ^]^

**Figure 7:**
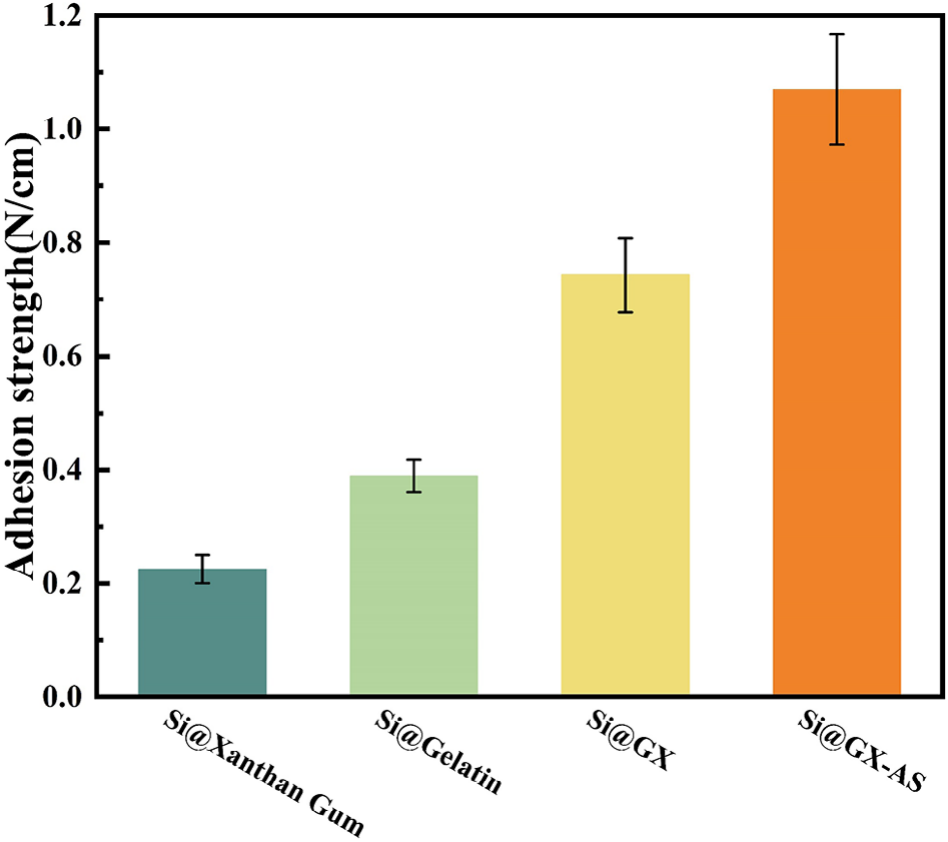
Adhesion of silicon anode foils prepared with different binders.

The adhesive strength of the binder is a critical physical property that affects the interfacial stability between the SiNPs and the current collector, thereby determining the cycling performance of the silicon anode.^[^ ^47^ ^]^ To evaluate the adhesive strength of different binders, the 180° peel test was used, and the results are shown in **Figure** **7**. First, the adhesive strength of Si@Gelatin was 0.389 ± 0.029 N cm^−1^. This is mainly due to the shorter chain length of gelatin, which leads to poor shear deformation resistance and easy breakage of anchor points under high stress.^[^ ^45^ ^]^ On the other hand, xanthan gum has poor rheological properties and dispersibility, making it difficult to form good infiltration with SiNPs,^[^ ^48^ ^]^ thus reducing the contact area and adhesive force between the binder material and SiNPs. Therefore, the adhesive strength (0.225 ± 0.025 N cm^−1^) is significantly lower than that of other samples.

The adhesive strength of Si@GX was 0.743 ± 0.066 N cm^−1^, higher than that of traditional binders such as PVDF and polyacrylic acid (PAA) (Figure S8, Supporting Information). This enhancement was attributed to the helical‐entangled network structure formed between gelatin and xanthan gum. In this structure, the hydrogen bond sites on the surface of gelatin can form multiple contacts with SiNPs and copper foil, and the shear deformation force can be uniformly dissipated to each hydrogen bond site along the backbone of xanthan gum and the side chains of gelatin, thereby improving the adhesive performance of the binder. Additionally, the adhesive strength of the sample treated with AS immersion reached 1.070 ± 0.097 N cm^−1^. This may be attributed to the Hofmeister effect, which enhances the formation of hydrogen bonds and the helical‐entangled network structure within the binder,^[^ ^14^, ^49^ ^]^ thereby increasing the hydrogen bonding interactions and contact area at the binder‐copper foil interface.^[^ ^50^ ^]^

During the electrode preparation process, gelatin and xanthan gum, as natural polymers with abundant polar functional groups, fully dissolved in water and formed hydrogen bonds with SiNPs and copper foil. Through helical recombination and Hofmeister salt solution immersion treatment, this interaction was further enhanced, ensuring tighter bonding and maintaining the integrity of contact between the active material, conductive agent, and current collector. Even after multiple folds (Figure S9, Supporting Information), no significant delamination was observed, indicating that GX‐AS can provide excellent mechanical properties and electrode integrity.

**Figure 8:**
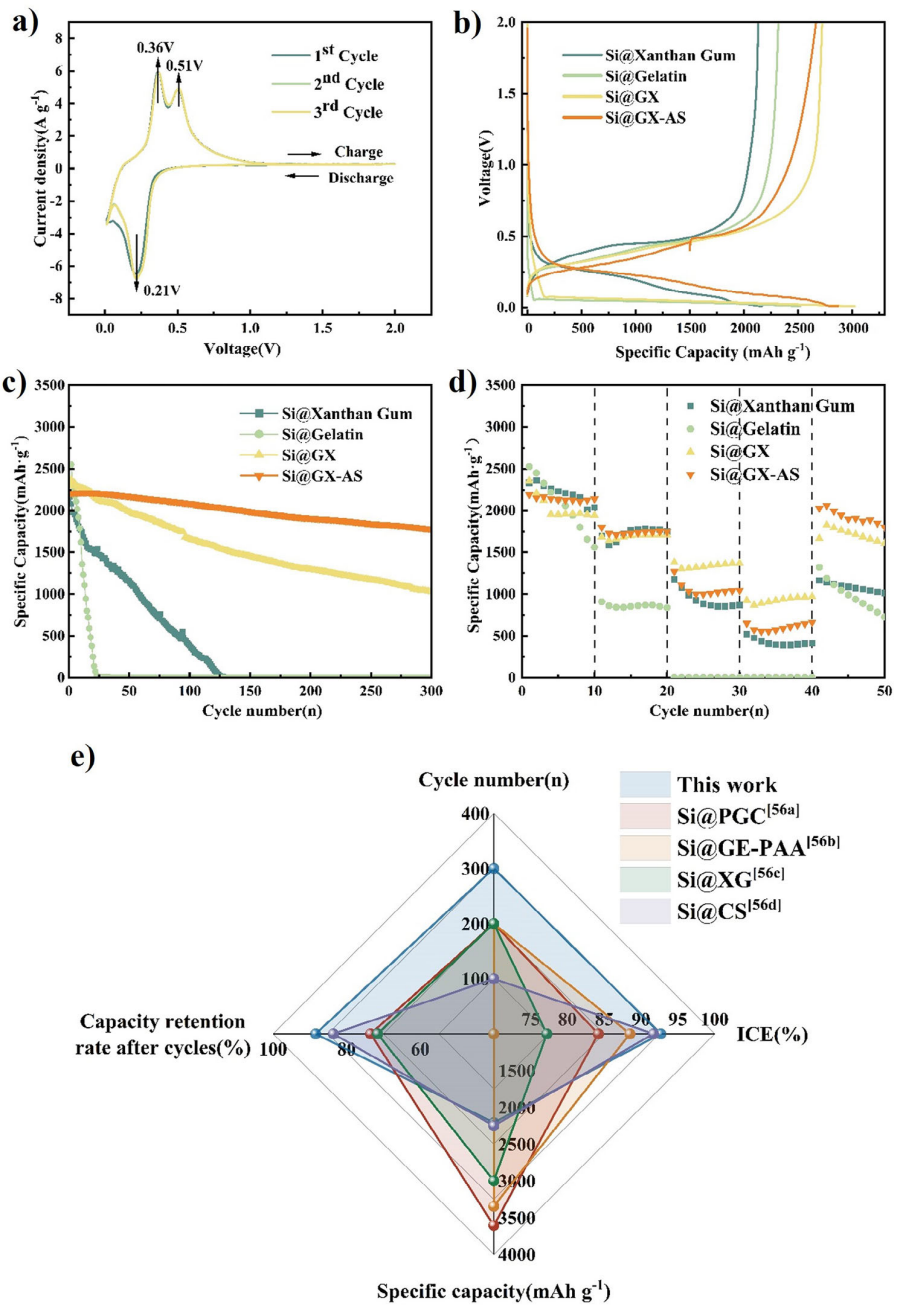
a) CV curves of the Si@GX‐AS anode at the scan rate of 0.1 mV s^−1^; b) Comparison of initial charge–discharge plots of Si anodes at 0.5 A g^−1^ for different binders;c) The cycling performance of silicon anode at 0.5 A g^−1^ for different binders; d) Rate performance of the Si electrodes with xanthan gum, gelatin, GX and GX‐AS binders at different current densities in the range of 0.1–1 C; e) comparison of electrochemical performance indices (initial Coulombic efficiency, initial specific capacity, number of cycles and capacity retention after cycles) with those reported in other literature.

To investigate the impact of different binders on the electrochemical performance of silicon anodes, cyclic voltammetry (CV), EIS, and Fick's law were used to analyze the electrochemical characteristics of the batteries. As illustrated in **Figure** **8**a, the CV curves reveal that the Si@GX‐AS sample displays a lithiation reduction peak at ≈0.21 V and oxidation peaks at 0.36 and 0.51 V, which correspond to the transition from amorphous LiSi to amorphous Si.^[^ ^51^ ^]^ Additionally, a faint reduction peak at ≈1.06 V was observed in the magnified section of the CV curve for the first cycle (Figure S10, Supporting Information), indicating electrolyte decomposition and the formation of the SEI.^[^ ^52^ ^]^

Figure 8b illustrates the performance of silicon anodes with different binders during constant current charge–discharge cycles, with initial coulombic efficiencies (ICE) of 73.88%, 81.12%, 88.25%, and 92.70% for Si@Xanthan Gum, Si@Gelatin, Si@GX, and Si@GX‐AS, respectively. Si@GX‐AS exhibited a high ICE, demonstrating that the helical‐entangled network could effectively suppress and buffer the volume expansion of silicon, maintain the integrity of the electrode surface, prevent side reactions with the electrolyte, minimize the formation of the SEI layer, and reduce Li^+^ consumption. In addition, after immersion in the AS solution, the initial specific capacity of Si@GX‐AS slightly decreased compared to Si@GX. This phenomenon might be attributed to the salting‐out effect promoting the helical‐entanglement of the GX‐AS molecular chains, resulting in a denser electrode network structure. Consequently, this reduced the number of active lithium reaction sites, leading to a decrease in the initial specific capacity.^[^ ^53^ ^]^

To compare the effects of different binders on the cycling performance of silicon anodes, cycling stability tests on half‐cells were conducted at a current density of 0.5 A g⁻¹. As shown in Figure 8c, the specific capacities of silicon anodes with different binders exhibited dramatic differences. The specific capacity of the Si@Gelatin anode dropped sharply after 20 cycles, from 2519.3 to 250.8 mAh g⁻¹. This decline is likely due to the insufficient length of gelatin molecular chains to effectively anchor SiNPs, leading to the fracture and detachment of SiNPs during cycling, which in turn causes the failure of the electrode structure. The Si@Xanthan Gum anode also exhibited significant capacity decay after 128 cycles, decreasing from 2165.7 to 14.4 mAh g⁻¹. This may be attributed to the rod‐like helical structure of xanthan gum molecular chains, lacking sufficient elasticity to buffer the drastic volume changes of silicon over the long term, resulting in rapid capacity decline in the later stages.^[^ ^54^ ^]^ To optimize the cycling stability of Si@GX, the mass ratio of gelatin to xanthan gum in GX was adjusted from 2:1 to 6:1 to determine the optimal ratio (Figure S11, Supporting Information), with the Si@GX51 anode showing the best cycling stability. Furthermore, the Si@GX51 anode foils were immersed in Hofmeister salt solutions with different anions, thoroughly dried, and then assembled into cells to test their cycling performance (Figure S12, Supporting Information). The results indicated that the cycling performance of the cells improved significantly with the enhancement of the salting‐out effect. Ultimately, the Si@GX‐AS anode, with a gelatin to xanthan gum mass ratio of 5:1 and immersed in AS solution, demonstrated an initial capacity of 2206.7 mAh g⁻¹ and maintained a specific capacity of 1779.8 mAh g⁻¹ after 300 cycles, achieving a capacity retention rate of 80.65%. This improvement is likely due to the increased helical entanglement of the protein‐like spatial structure formed by the recombination of gelatin and xanthan gum after being immersed in AS solution. This enhanced helical‐entangled network better buffers and releases the stress and strain changes caused by the volume expansion of silicon, maintaining the integrity of the network structure and significantly improving the cycling life of the silicon anode.

To evaluate the effect of different binders on the rate capacity of silicon anodes, Figure 8d illustrates the change in specific capacity of the half‐cell as the current density varies from 0.1 to 1C (1C = 4200 mAh g^−1^) and then returns to 0.1C. It can be observed that at a current density of 0.1C, the specific capacity of Si@Gelatin decreased from 2525.6 to 1562.2 mAh g^−^¹, and at 0.5C, the specific capacity approached 0, which is consistent with its cycling performance test results. Additionally, the specific capacities of other silicon anodes also decrease with increasing current density. When the current density reached 1C, Si@GX and Si@GX‐AS still maintained specific capacities of 925.5 and 649.7 mAh g^−1^, respectively. Furthermore, when the current density is restored to 0.1C, Si@GX‐AS recovers to 92.6% of its initial specific capacity, while Si@Xanthan Gum, Si@Gelatin, and Si@GX exhibit significantly lower recovery rates. This indicates that Si@GX‐AS exhibits excellent rate performance, which may be attributed to its helical‐entangled network structure. This structure not only provides mechanical stability but also maintains the integrity of the network structure during cycling, preserving the porosity within the network. This allows Li^+^ to pass through quickly and reduces their diffusion resistance within the electrode material, thereby ensuring that the electrode retains good electrical conductivity and ion transport performance even at high current densities.

Compared with traditional binders such as PVDF and PAA, the new binders exhibited superior electrochemical performance (Figure S13, Supporting Information). Furthermore, compared to other reported binder materials including natural polymers, synthetic polymers, and composites with functional network structures,^[^ ^55^ ^]^ it is discovered that the Si@GX‐AS silicon anode has a higher ICE, more stable cycling performance (Figure 8e), and more comparative data can be found in Table S1 (Supporting Information). The primary reasons for this enhanced performance are the presence of numerous polar functional groups in the new binders, which improves the interaction with SiNPs, and the helical‐entangled network structure, which enhances the structural stability of the anode.

**Figure 9:**
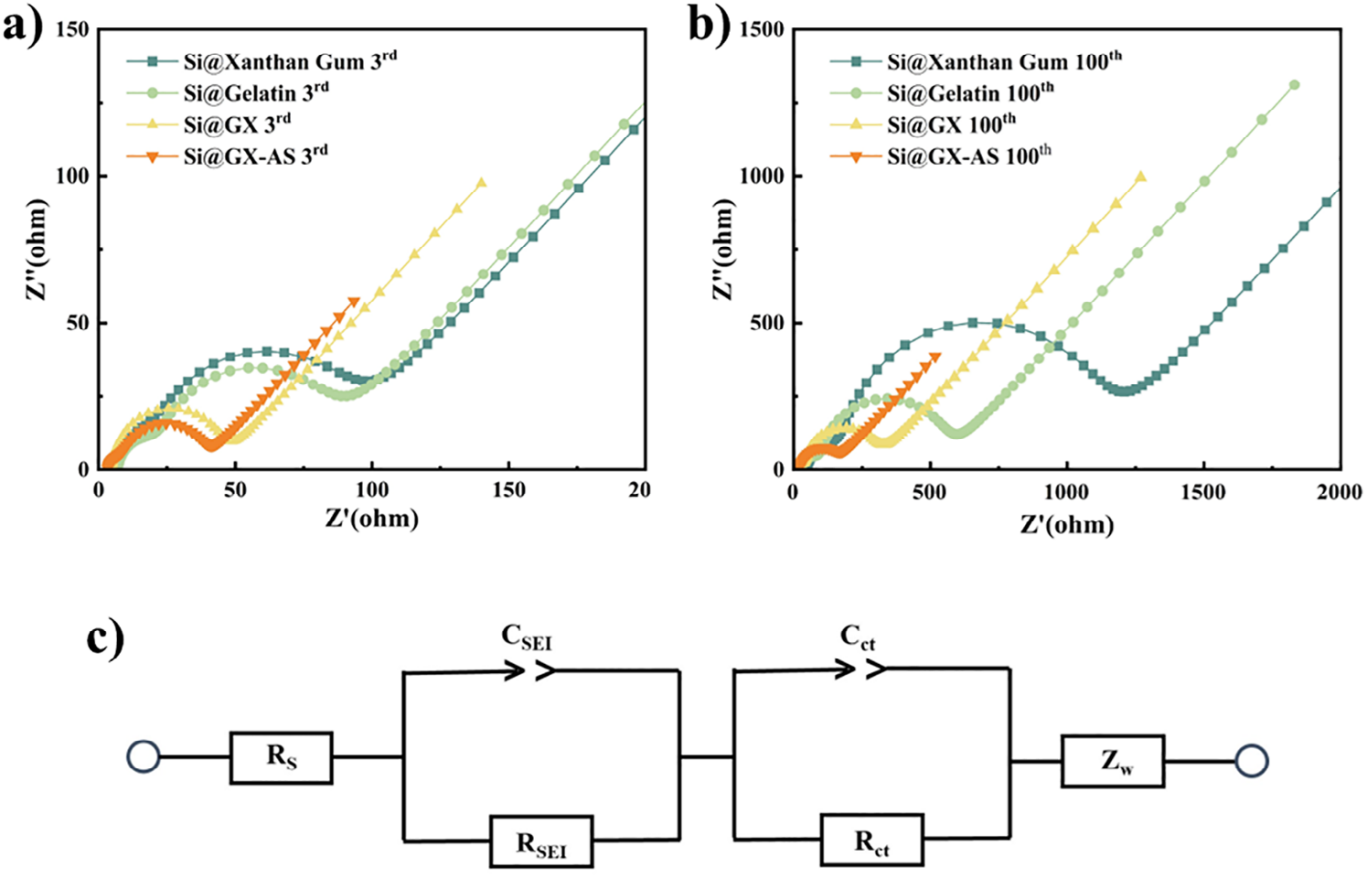
Nyquist plots of different Si anodes after 3 cycles a) and 100 cycles b); the equivalent circuit model c).

To further investigate the optimization mechanism of the electrochemical performance of Si@GX‐AS electrodes, this study used EIS to compare the interfacial resistance of Si@Xanthan Gum, Si@Gelatin, Si@GX, and Si@GX‐AS electrodes after the 3rd and 100th cycles. **Figure** **9** shows the Nyquist plots and corresponding equivalent circuit models obtained from the tests. From the Nyquist plots, it can be seen that in the high‐frequency and mid‐frequency regions, all the electrodes exhibit depressed semicircles, which represent the contributions of solid‐electrolyte interface resistance (R_SEI_) and charge transfer resistance (R_ct_), respectively. In the low‐frequency region, all electrodes show linear characteristics with decreasing slopes, reflecting the diffusion process of lithium ion (Li^+^) within SiNPs, i.e., the influence of Warburg impedance (Z_w_).^[^ ^56^ ^]^ As shown in Figure 9a, after 3 cycles, the Si@GX‐AS electrode has the smallest semicircle diameter in the mid‐frequency region, indicating the smallest R value. **Table** **2** lists the R_SEI_ and R_ct_ values of each electrode after the 100th cycle, showing that the Si@GX‐AS electrode still has the lowest R (R_SEI_ = 5.24Ω, R_ct_ = 14.83Ω). The results indicate that the GX‐AS binder can effectively maintain the stability of the SEI layer and the continuity of the electronic conduction. To further investigate the diffusion behavior of lithium ions in the solid phase, this study used EIS to calculate the lithium‐ion diffusion coefficient ($D_{Li^{+}}$) based on the semi‐infinite diffusion impedance model and the derivation of Fick's first and second laws, with the specific formulas as follows:

(1) $$D_{Li^{+}}=\frac{R^{2}T^{2}}{2A^{2}n^{4}F^{4}C^{2}\sigma^{2}}$$

**Table 2:** Impedance of Si anode with different binders and $D_{Li^{+}}.$.

Sample	R_SEI_ [Ω]	R_ct_ [Ω]	σ [Ω·cm^2^ mol^−1^]	D_Li+_ [cm^2^ s^−1^]
Si@Xanthan Gum 3rd	5.36	62.17	166	8.84 × 10^−16^
Si@Xanthan Gum 100th	46.20	886.40	328	4.35 × 10^−18^
Si@Gelatin 3rd	6.67	54.91	351	1.13 × 10^−15^
Si@Gelatin 100th	26.78	60.59	1164	9.31 × 10^−16^
Si@GX 3rd	4.48	37.91	77	9.14 × 10^−15^
Si@GX 100th	6.76	240.30	249	8.82 × 10^−16^
Si@GX‐AS 3rd	3.28	4.98	50	2.17 × 10^−14^
Si@GX‐AS 100th	5.24	14.83	97	5.79 × 10^−15^

In the formula, (R) represents the universal gas constant, 8.314J mol^−1^k^−1^; (T) is the absolute temperature, 298.15 K; (A) is the area of the electrode material; (n) is the number of electrons transferred; (F) is the Faraday constant, 96486C mol^−1^; (C) is the Li^+^ concentration; and (σ) is the Warburg coefficient, which can be obtained from the slope of the low‐frequency region of the Nyquist plots.^[^ ^57^ ^]^

The D_Li+_ values can be calculated using the expression (1), and Table 2 presents the D_Li+_ values for each electrode. It can be observed that the D_Li+_ value of the Si@GX‐AS electrode is significantly higher than that of the other electrodes, indicating a higher Li^+^ conductivity.^[^ ^58^ ^]^

**Figure 10:**
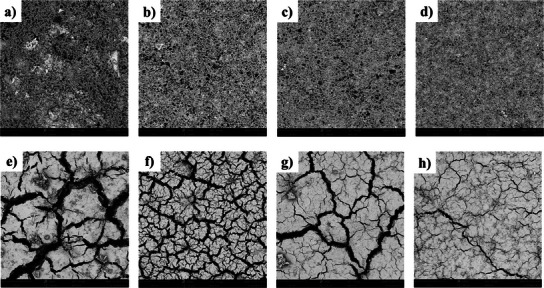
Top‐view of FESEM surface morphology of electrode materials prepared with different binders before and after 100 cycles: a,e) Si@Xanthan Gum; b,f) Si@Gelatin; c,g) Si@GX; d,h) Si@GX‐AS.

To verify the effectiveness of the helical‐entangled network structure in maintaining electrode integrity, the morphological changes of different silicon anodes before and after 300 charge–discharge cycles were observed through Field‐Emission Scanning Electron Microscope (FESEM). As shown in **Figure** **10**a–h, all silicon anodes exhibited good structural integrity before cycling. However, dramatic differences were observed on the surfaces of silicon anodes with different binders after 300 cycles. Specifically, varying degrees of crack formation were observed on the surfaces of the four samples, with the cracks on the Si@Xanthan Gum being the most pronounced. In contrast, the Si@GX‐AS sample, which underwent salt soaking treatment, exhibited only a few cracks.

**Figure 11:**
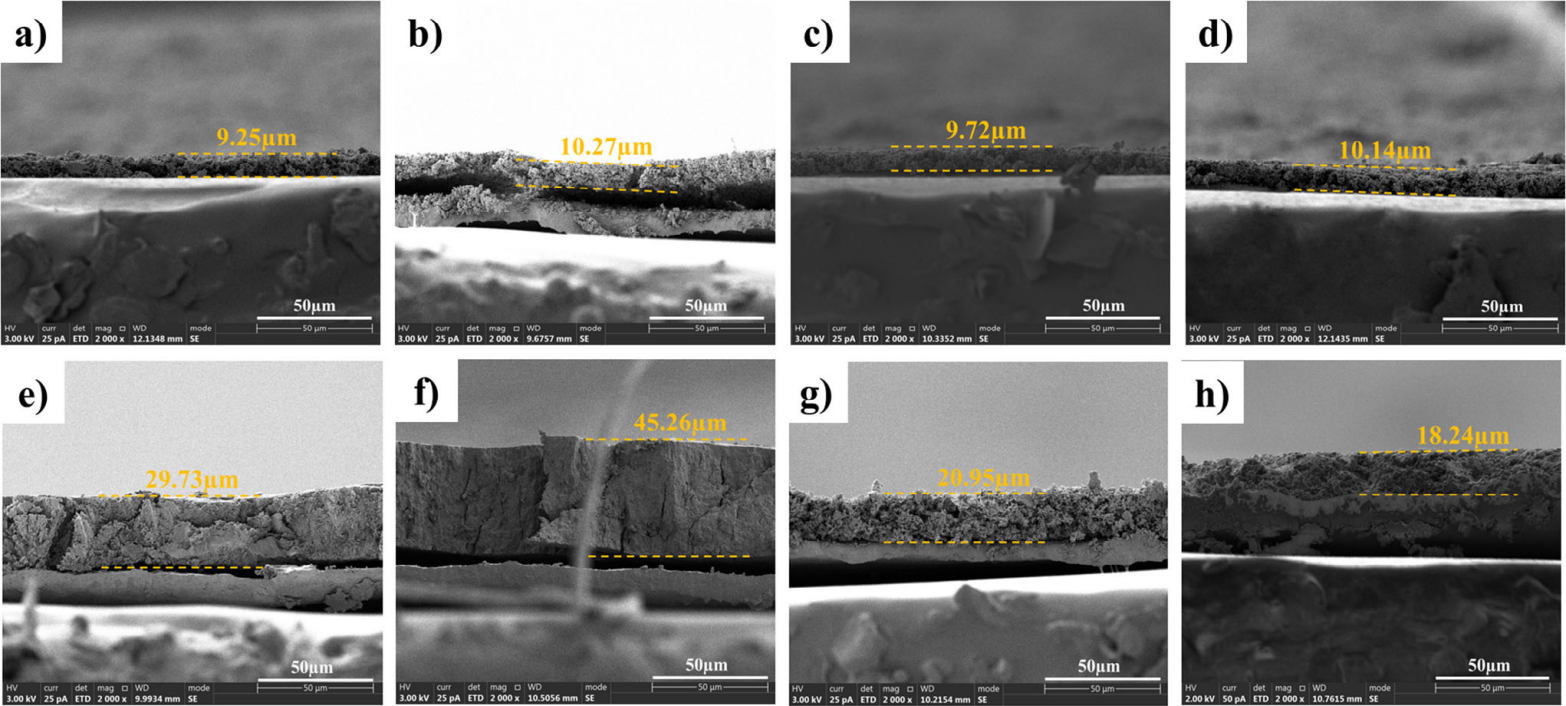
Cross‐sectional FESEM images of electrode materials prepared with different binders before and after 100 cycles: a,e) Si@Xanthan Gum; b,f) Si@Gelatin; c,g) Si@GX; d,h) Si@GX‐AS.

Additionally, FESEM was also employed to examine the cross‐sectional alterations in diverse silicon anodes before and after the cycle. As illustrated in **Figure** **11**a–h, the initial thicknesses of the Si@Xanthan Gum, Si@Gelatin, Si@GX, and Si@GX‐AS samples are 9.25, 10.27, 9.72, and 10.14 µm, respectively. After 100 cycles, the cross‐sectional thicknesses changed to 29.73, 45.26, 20.95, and 18.24 µm, respectively, and the expansion rates of the four polar plates are 321.41, 440.70, 215.53, and 179.88%, respectively. From the top view and cross‐sectional images of Si@GX‐AS, it can be observed that the optimized helical‐entangled network structure effectively suppresses and buffers the volume change of Si, thereby maintaining the integrity of the electrode.

**Figure 12:**
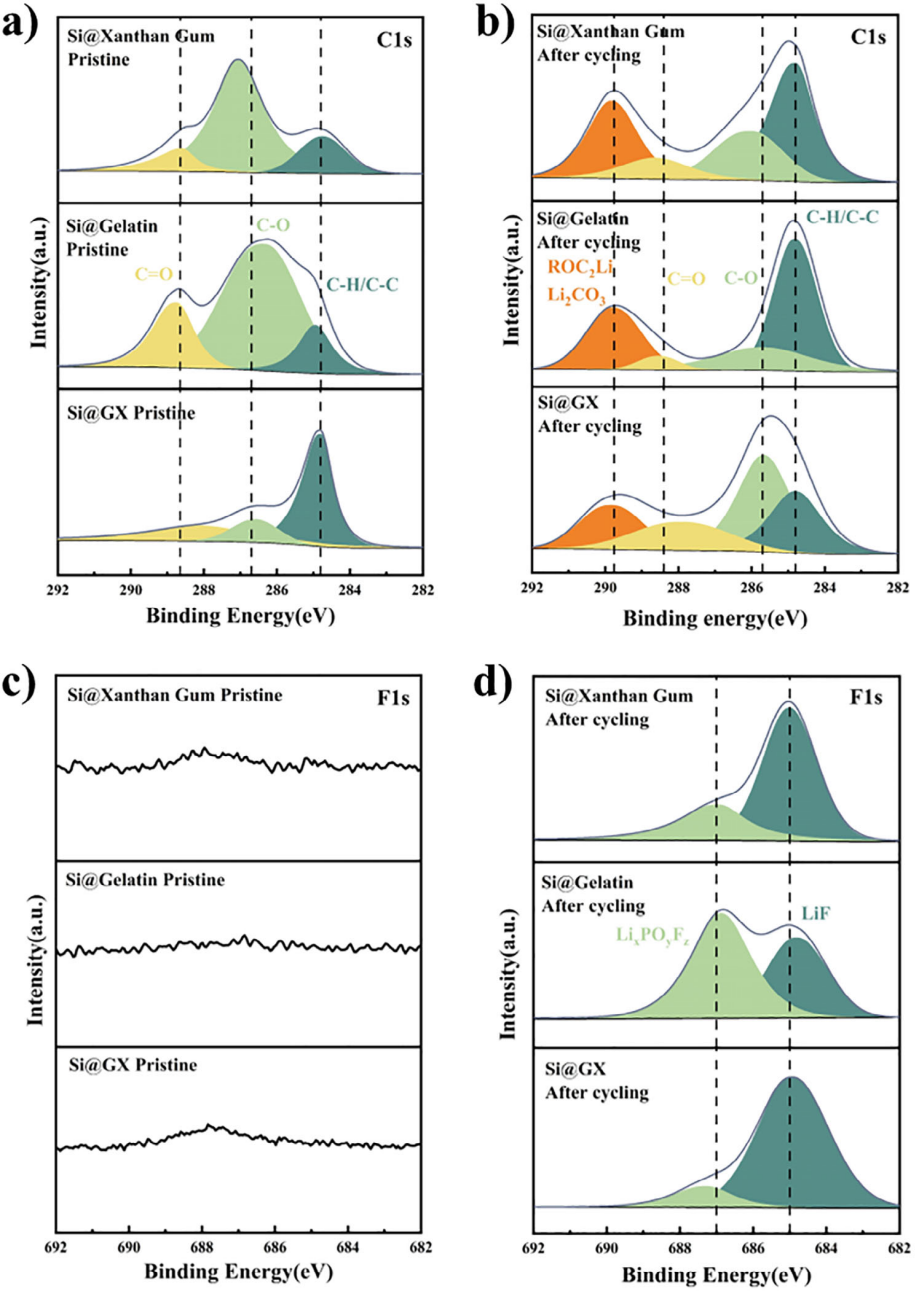
XPS spectra of C1s a,b) and F1s c,d) were obtained from Si@Xanthan Gum, Si@Gelatin, and Si@GX before and after 100 cycles.

To investigate the stability of the SEI, a detailed analysis of the chemical composition of the SEI layer was conducted using X‐ray photoelectron spectroscopy (XPS). **Figure** **12** illustrates the XPS spectra of a silicon anode with xanthan gum, gelatin, and GX as binder, both before and after the cycling process. As illustrated in Figure 12, the C1s spectrum of the electrode prior to cycling reveals the presence of characteristic peaks associated with C─H/C─C, C─O, and C═O at 284.8, 286.7, and 288.6 eV,^[^ ^59^ ^]^ respectively. Following 100 cycles, the C1s spectrum exhibited the emergence of a new peak at 288.96 eV, which is attributed to the decomposition products of the electrolyte, namely ROCO_2_Li and Li_2_CO_3_.^[^ ^60^ ^]^ Additionally, the F1s spectral analysis revealed the presence of characteristic peaks associated with Li_x_PO_y_F_z_ and LiF at 687.1 eV and 685.0eV,^[^ ^61^ ^]^ respectively. A comparison of the C1s and F1s spectra of the three binders reveals that the intensity of the ROCO_2_Li, Li_2_CO_3_ and Li_x_PO_y_F_z_ peaks formed by the Si@GX electrode is lower than that of the Si@Xanthan Gum and Si@Gelatin electrodes. This indicates that the decomposition products of the Si@GX electrolyte are less significant. This finding demonstrates that the GX‐AS binder can facilitate the formation of a more stable and LiF‐rich SEI layer on the surface of the silicon negative electrode, effectively inhibiting the continuous decomposition of the electrolyte and thereby markedly enhancing the cyclic stability of the electrode. By analyzing the N1s spectra of Si@Gelatin anodes before and after cycling (Figure S14, Supporting Information), it was found that a characteristic peak existed at 399.6 eV both before and after the reaction.^[^ ^62^ ^]^ This characteristic peak was mainly attributed to free amine groups, indicating that the amine groups of gelatin did not decompose during the reaction. Additionally, the slight shift in the characteristic peak might be due to charge transfer between the nitrogen atoms and surrounding atoms during the reaction, thereby affecting the binding energy.^[^ ^63^ ^]^

**Figure 13:**
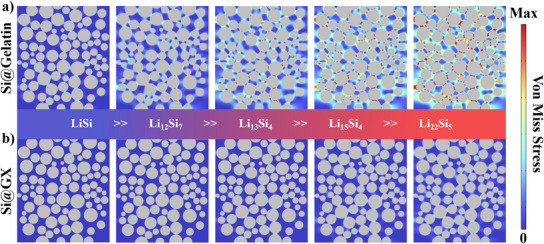
Stress evolution of the a) Si@Gelatin and b) Si@GX electrodes at different lithiation states, simulated by the finite element method.

In order to examine the distribution of stress experienced by different binder materials during the removal of lithium from silicon anodes, a finite element analysis was conducted using the COMSOL Multiphysics 5.4 software. As illustrated in **Figure** **13**, the two binder materials display disparate stress distributions as a consequence of their distinct mechanical properties. The insertion of Li^+^ results in the continued expansion of the silicon nanoparticles. The short‐chain structure of gelatin is not conducive to the formation of a stable entangled network structure, which results in the expanded silicon particles breaking away from the gelatin network and squeezing and colliding with each other, thereby concentrating the stress. In contrast, the Si@GX samples exhibit a relatively low stress distribution even in the fully Li_22_Si_5_ state (Figure 13b). This may be attributed to the fact that the entangled network restricts the movement of silicon particles, reduces the frequency of direct collisions between silicon nanoparticles, and maintains the stability of the electrode. Furthermore, its helical structure acts as a soft buffer layer, which can alleviate the issue of stress concentration. Consequently, Si@GX is more effective in maintaining the structural integrity of the electrode throughout the cycle.

## 3 Conclusion

In this study, we successfully developed a gelatin‐xanthan gum binder with a protein‐like spatial structure through the synergistic effects of the thermo‐responsive reorganization of molecular chains and the salt‐out effect of the Hofmeister sequence. Similar to natural protein structures, GX‐AS effectively transmits the stress and strain caused by silicon expansion to the helical regions through its helical‐entangled spatial structure. These regions absorb and dissipate stress through repeated bending, twisting, and stretching, ensuring the long‐term stability of the electrode structure. The silicon anode using the GX‐AS binder exhibited high initial Coulombic efficiency, specific capacity, rate performance, and cycling stability. This innovative approach highlights the potential of natural polymers to form complex 3D structures, providing a new direction for designing high‐performance silicon anode binders. Additionally, this new method can be used to prepare flexible and foldable electrode materials, expanding its application potential in wearable devices and health monitoring fields.

## 4 Experimental Section

### Materials

SiNPs with a diameter of 80 nm were purchased from the Nanhao Electronic Technology Company (Anhui, China). Gelatin (gel strength ≈300 g Bloom), PVDF, and PAA were purchased from Sigma–Aldrich. Xanthan gum was purchased from Aladdin. AS (>99%) was purchased from Sinopharm Chemical Reagent Co., Ltd. The commercial electrolyte and Super P carbon black were purchased from Taiyuan Lizhiyuan Technology Co., Ltd. The lithium foil was sourced from Wuxi Yiri New Materials Co., Ltd. The above reagents could be used directly without further purification.

### Synthesis of GX

Gelatin was dissolved in deionized water to prepare a 5% gelatin solution, which was heated in a water bath at 80 °C for 15 min until the gelatin was completely dissolved and the solution was bubble‐free. The corresponding proportion of xanthan gum was added to the gelatin solution, and the mixture was heated and stirred in a water bath at 80 °C for 30 min until the solution was homogeneous. The mass ratio of gelatin to xanthan gum was adjusted from 2:1 to 6:1, and the prepared samples were designated as GX21, GX31, GX41, GX51, and GX61.

### Electrode Material Preparation

SiNPs, binder, and conductive agent Super P were mixed in a mass ratio of 8:1:1, added with deionized water, and stirred in a water bath at 80 °C for 30 min until the slurry was uniform. The slurry was then coated on the copper foil, which was expressed as Si@GX. Si@GX was placed in a refrigerator at 5 °C for 8 h, then soaked in the 12% ammonium sulfate solution for 12 h and dried in a vacuum drying oven at 80 °C for 12 h. The obtained electrode sheet was recorded as Si@GX‐AS.

### Battery Assembly

The coin cells (CR2025) used lithium foil as the counter electrode, and the prepared silicon anode foil was punched into a 16 mm diameter disk as the working electrode. The separator (Celgard 2400) and 75 µL of electrolyte were used. The electrolyte consisted of 1 m LiPF6 in a 1:1:1 volume ratio mixture of ethylene carbonate, diethyl carbonate, and dimethyl carbonate. The loading mass of SiNPs was determined by calculating the mass ratio of the slurry components once the copper foil was completely dried, prior to immersion in the salt solution, and was ≈1.2 mg cm⁻^2^. The coin cells were assembled in an argon‐filled glove box. To more comprehensively evaluate the electrochemical performance of the new binder, PVDF and PAA were also used as binders for comparison.

### Materials Characterization

Rotational rheometers (DHR‐2), viscometers (SNB‐4), and DSC (TA250) were employed to investigate the glass transition and thermal denaturation temperatures of the binder. The chemical composition and interactions within Si anodes were characterized using FTIR (Bruker Vertex 70) and XPS (Thermo Scientific 250Xi). Gelatin, xanthan gum, GX, and GX‐AS were analyzed via XRD (Bruker D8 Advance). The surface morphology and cross‐sectional thickness of Si@GX were examined with a FESEM (Thermo Scientific Apreo 2). To characterize the mechanical properties of binder materials prepared with varying ratios of xanthan gum and gelatin, compression tests were first conducted on cylindrical hydrogels (diameter 30 mm, height 30 mm). Next, tensile tests were performed on the dried binder films, which were cast into strips of 100 mm × 10 mm × 0.5 mm, with a tensile speed of 100 mm min^−1^. Finally, the adhesive strength of the binder was assessed through a 180° peel test on the electrode sheet, with a sample width of 19 mm, a peel speed of 20 mm min^−1^, using 3M  Scotch 600 tape. All tests were conducted using an Instron Tester 5982.

MD simulations were conducted using the Forcefield‐based Simulation Tool (Forcite) within the Materials Studio program to elucidate the entanglement of polymer chains within the hydrogel. The PAA hydrogel model, comprising PAA long chains with 50 monomers: epichlorohydrin: H_2_O in a ratio of 10:8:1500 and xanthan gum chains with 12 monomers: gelatin chains with 3 monomers: H_2_O in a ratio of 8:15:1200, was packed into a periodic simulation box with a density of 1.2 g cm⁻^3^. The system underwent energy minimization using the steepest descent algorithm with a force tolerance of 0.005 kcal mol⁻¹ Å⁻¹. Subsequently, the system was equilibrated under the NPT ensemble for a relaxation process for 2 ns at 298 K and 1 bar, employing a time step of 1 fs. To eliminate rigidity effects, an annealing process was performed on the hydrogel box, with the temperature ramped between 300 and 700 K over a duration of 6 ns. Following equilibration, production MD simulations were carried out for 12 ns under identical conditions. To simulate experimental conditions more closely, the MD simulations of xanthan gum‐gelatin hydrogels included a multi‐step process: holding the system at 298 K for 4 ns, raising the temperature to 353 K for 4 ns, and subsequently cooling it back to 298 K for an additional 4 ns, thereby completing the simulation process.

### Electrochemical Testing

The Si anodes were subjected to galvanostatic charge–discharge tests using a battery tester (LANHE, CT3002A) in the voltage range of 0.01–2 V versus Li/Li^+^. The current density for the cycling test was maintained at 500 mA g^−1^, while for the rate test, it was changed from 0.1 to 1C. EIS tests were conducted by applying a sinusoidal signal with a 10 mV amplitude over a frequency range of 0.01–100 kHz using an electrochemical workstation (CHI,660E). CV tests were performed using an electrochemical workstation (Princeton, PA4000) with a voltage of 0.01–2 V at a scan rate of 0.1 mV s^−1^.

## Conflict of Interest

The authors declare no conflict of interest.

## Supporting information



Supporting Information

## Data Availability

Research data are not shared.
