# Supplementary material for: Construction of Protein‐Like Helical‐Entangled Structure in Lithium‐Ion Silicon Anode Binders via Helical Recombination and Hofmeister Effect
Source: Adv Sci (Weinh). 2025 Apr 27;12(20):2412769. doi: 10.1002/advs.202412769 (PMC12120714; doi:10.1002/advs.202412769)
Supplement: Supplementary file 1 — Supporting Information [file ADVS-12-2412769-s001.docx]

Supplementary information

**Construction of Protein-like Helical-Entangled Structure in Lithium-Ion Silicon Anode Binders via Helical Recombination and** **Hofmeister Effect**

Shiyuan Dai^1,2^, Fei Huang^2^, Jinglun Yan^2^, Yuan Yuan Sun^3^, Chao Chen^2,4,5*^, HaiDong Li^2*^

^1^School of Materials Science and Engineering, Zhejiang Sci-Tech University, Hangzhou, China

^2^Nanotechnology Research Institute, Jiaxing University, Jiaxing, China

^3^College of Chemistry and Chemical Engineering, Yangzhou University, Yangzhou, China

^4^G60 STI Valley Industry & Innovation Institute, Jiaxing University, Jiaxing, China

^5^Shanghai Institute of Applied Physics, Chinese Academy of Science

*****Correspondence: [chaochen@zjxu.edu.cn,](mailto:chaochen@zjxu.edu.cn,) hdlipr@163.com


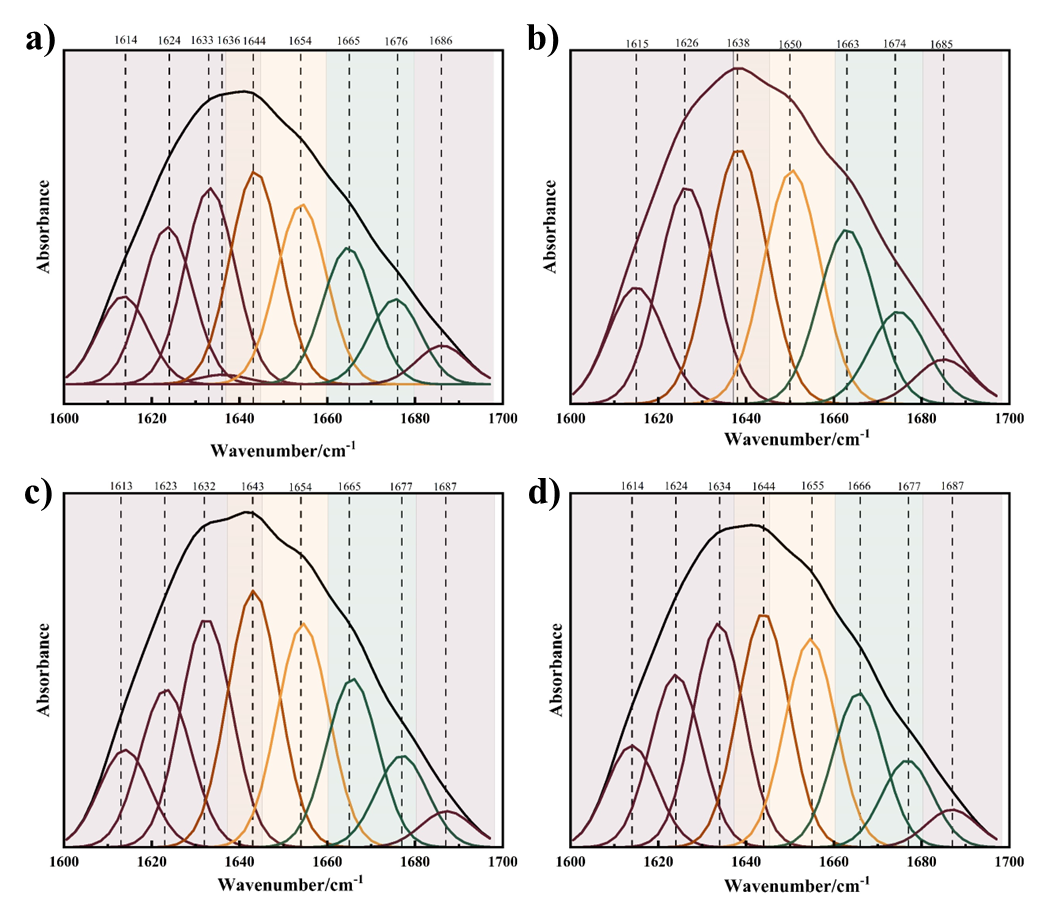


Figure S1. IR deconvolution fits for xanthan gum(a), gelatin(b) , GX(c) , GX-AS (d) amide I band


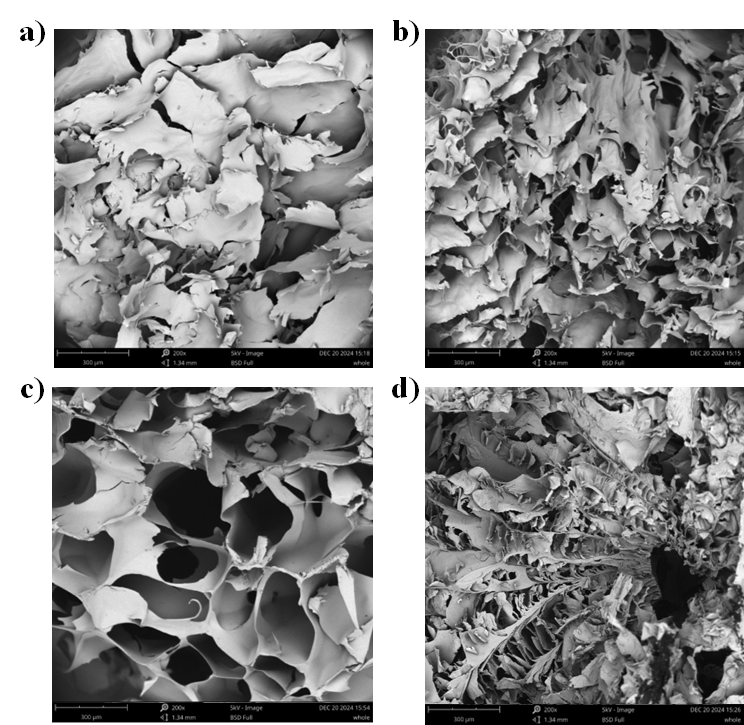


Figure S2. Cross-sectional SEM images of freeze-dried binder samples: a) xanthan gum; b) gelatin; c) GX; d) GX-AS

Compression modulus tests were conducted on gelatin and GX hydrogels with varying ratios. As the mass ratio of gelatin to xanthan gum varied from 2:1 to 6:1, GX51 exhibited the highest compression strain and modulus (Figure S3). The supposed reason was that at a 5:1 mass ratio of gelatin to xanthan gum, the rigid double-helix structure of xanthan gum and the flexible triple-helix structure of gelatin form optimal hydrogen and ionic bonds. These interactions help form a uniform and stable network structure, thereby enhancing the mechanical properties of the composite material. Excessive gelatin may lead to its aggregation, forming uneven regions and reducing the mechanical properties of the composite. Conversely, insufficient gelatin may result in a less compact network structure, similarly affecting mechanical properties.


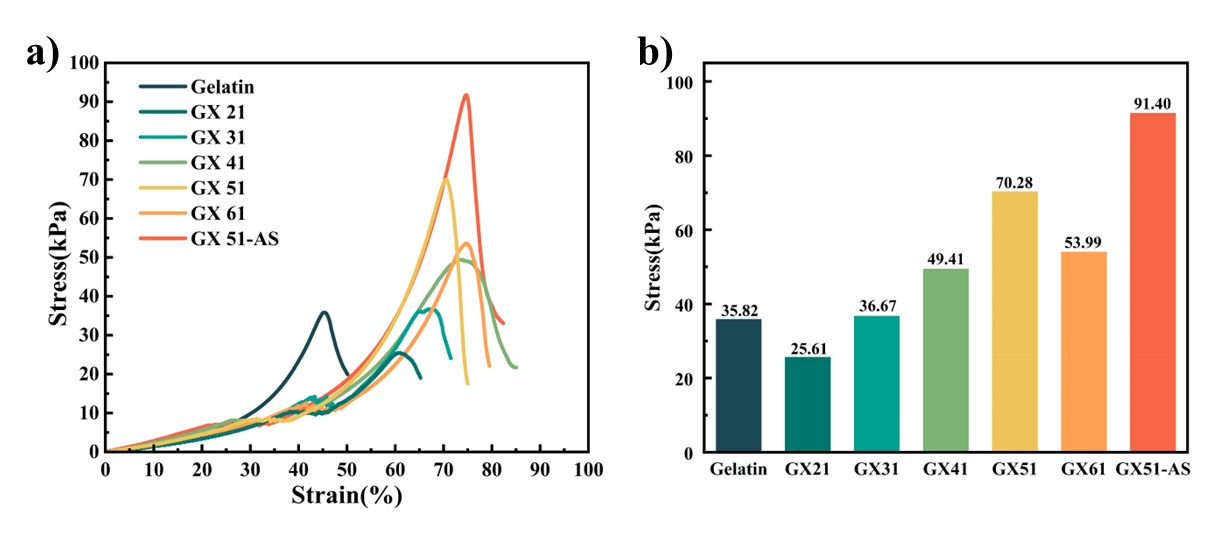


Figure S3. Stress-strain curves (a) and compressive Young's modulus (b) of gelatin and GX with different ratios

In the Hofmeister series, the influence of anions is typically more pronounced than that of cations. To systematically evaluate the effects of different anions on GX hydrogels, the GX hydrogels were immersed in ammonium chloride (NH_4_Cl), ammonium acetate (NH_4_Ac), and ammonium sulfate (AS) solutions for 12 hours. Following the removal of any residual salts from the surface, the compression modulus was then tested (Figure S4). The results showed that the compression modulus of the immersed GX hydrogels increased with the salting-out ability of the anions. The greatest increase in compression modulus was observed for GX-AS, which rose from 78.47 kPa prior to immersion to 112.08 kPa. In contrast, the salting-out abilities of acetate and chloride ions were weaker, so their impact on the compression modulus was minimal. This may be because anions with a strong salting-out effect (such as sulfate ions) can more effectively disrupt hydrogen bonds between water molecules, thereby reducing the solubility of gelatin and xanthan gum in water. This facilitates the closer proximity of polymer chains within the hydrogel, which then forms a more compact network structure. This consequently enhances the mechanical properties of the hydrogel.


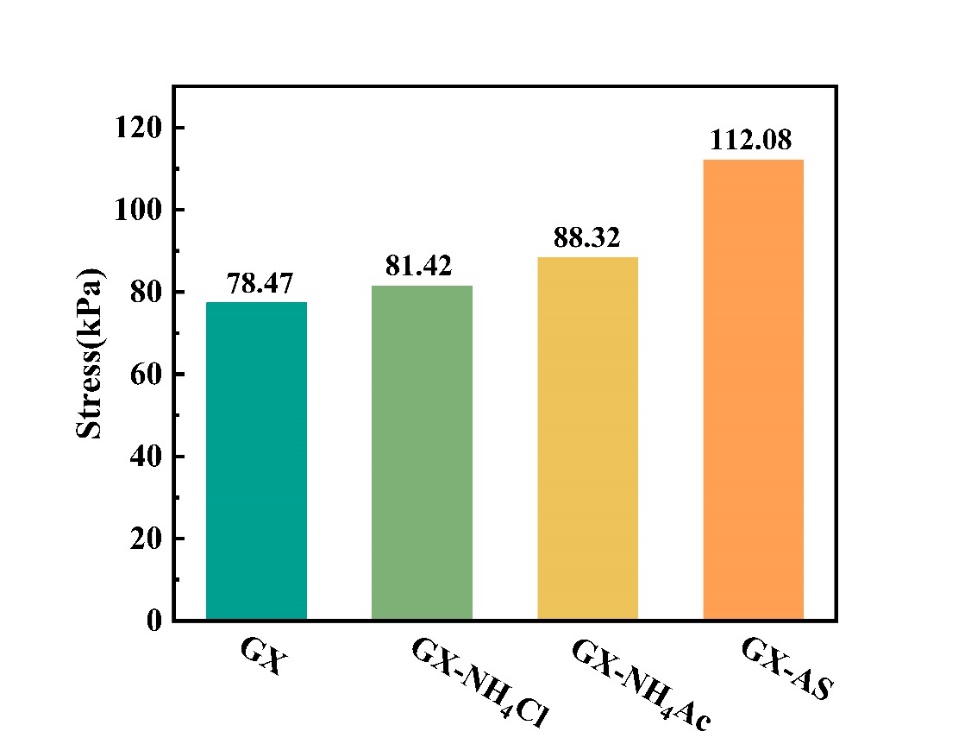


Figure S4. Mechanical properties of Si@GX gels after soaked in different Hofmeister salt solutions

15 cyclic compression tests were conducted on the GX hydrogel without soaking treatment. In comparison to GX-AS, the untreated GX hydrogel demonstrated a lower degree of hysteresis curve overlap. This may be attributed to the inadequate formation of a helical entangled network structure in the GX hydrogel that has not undergone Hofmeister salt soaking treatment. This results in a reduction in energy storage and release efficiency following compression, an increase in energy loss, and a decline in stability. Consequently, Hofmeister salt soaking treatment is crucial for enhancing the compactness of the helical entangled network structure and improving its structural stability.


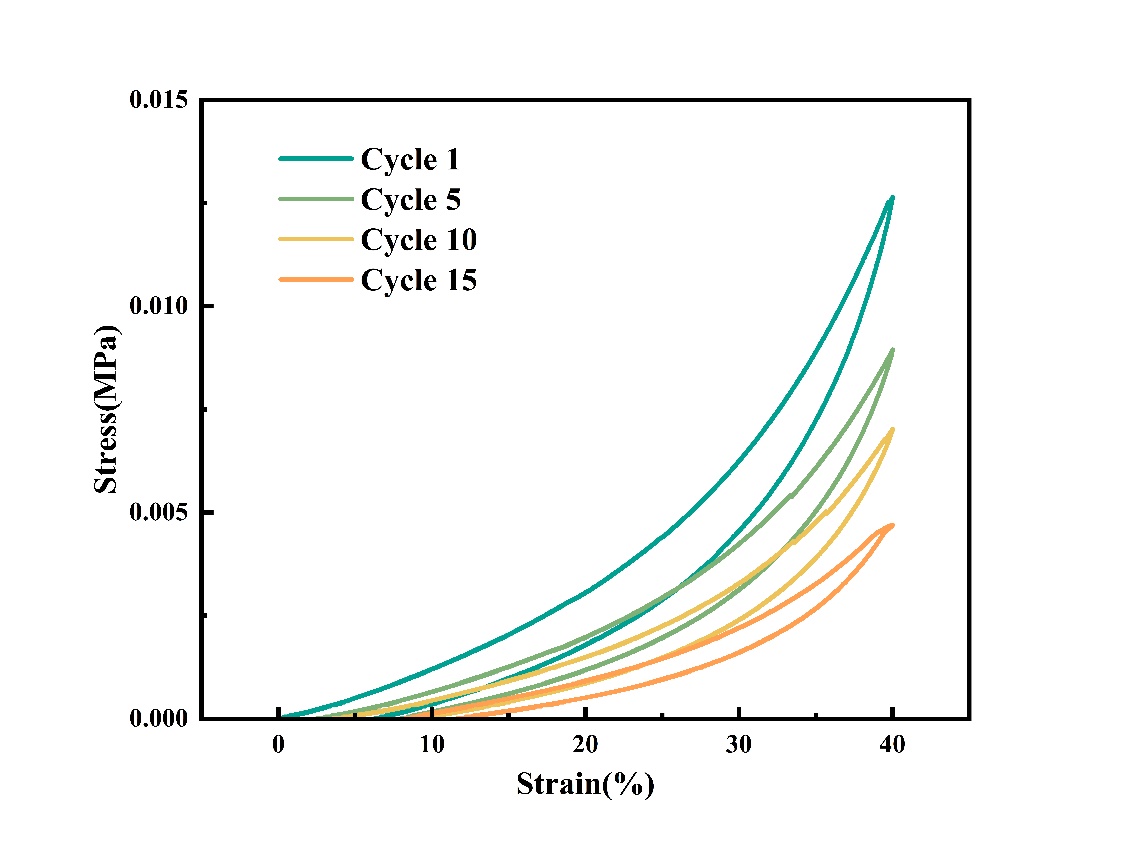


Figure S5. Fatigue resistance curve of GX under loading-unloading cyclic test

Figure S6 showed the stress-strain curves of dried films of xanthan gum, gelatin, GX, and GX-AS. Xanthan gum reached a stress of approximately 5 MPa at a strain of about 0.5%, after which the stress did not change significantly. This indicated that xanthan gum reached its yield point early during stretching, with its structure undergoing significant changes within a small strain range. The stress-strain curves of gelatin, GX, and GX-AS all exhibited a small change in stress during the initial strain stage (plateau region), followed by a continuous increase in stress. This phenomenon was related to the helical-entangled network of the molecular chains[1]. The untangling process of the helical segments required a large amount of energy to fully unfold, resulting in a small change in stress during this period, and the higher the degree of helical entanglement, the longer the duration of the plateau region. As the helical segments fully unfolded, the molecular chains began to be truly stretched, leading to a significant increase in stress [2]. Among them, GX-AS exhibited the highest stress value, exceeding 70 MPa at a strain of about 2.5%. This was mainly due to the treatment with ammonium sulfate solution, where the salting-out effect made the helical structure of the protein-like molecules more compact, thus dissipating more energy during the unwinding process. The compact helical structure also enhanced the hydrogen bonding interactions between GX-AS molecules, making it more difficult for the polymer chains to separate and slide when stretched [3]. Therefore, after the plateau region ended, the molecular chains of GX-AS required higher stress to be further stretched, resulting in higher tensile strength.


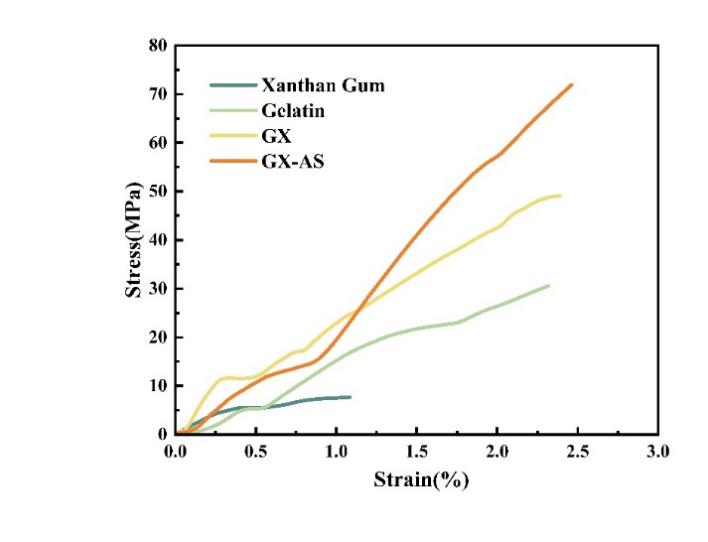


Figure S6. Stress-strain curves of dried films of xanthan gum, gelatin, GX, and GX-AS.

Through MD simulations, the structural characteristics of the traditional binder PAA and the xanthan gum-gelatin composite binder under an external force field were systematically explored. As shown in Figure S7a, when the crosslinked network of PAA, constructed using epichlorohydrin as the crosslinking agent, was subjected to an external force field at 298K, the interactions between molecular chains were relatively weak, and the arrangement of molecular chains became significantly loose after being stressed. After 12ns of simulation, this network showed a dissociation state, resulting in poor resistance to deformation. Figure S7b demonstrated the dynamic response behavior of the xanthan gum-gelatin composite binder network under an external force field and different temperature conditions. When operated for 12ns at 298K, the composite network showed a low degree of molecular chain entanglement and exhibited a loose network structure. However, when subjected to a heating-cooling cycle, the composite network exhibited significant dynamic reorganization ability. Initially operated at 298K for 4ns, the loosening of molecular chains remained evident; Upon increasing the temperature to 353K (80 ℃), the extended molecular chains became more flexible, and the intermolecular interactions dominated by hydrogen bonds between polar functional groups (such as hydroxyl, carboxyl, and amide groups) gradually enhanced, significantly shortening the distance between molecular chains, making the network structure become more compact and initially forming an entangled network[4]. Subsequently, after cooling to 298K and operating for another 4ns, the molecular chains of xanthan gum and gelatin tended to recover their helical conformations, which further enhancing the entanglement and interactions between molecular chains, resulting in the network structure exhibiting higher compactness and stability rather than loose dissociation under the external force field.

In summary, MD simulation results indicated that the xanthan gum-gelatin composite could achieve dynamic reorganization through thermal response, forming a stable helical-entangled network structure, effectively mitigating the loosening of molecular chains caused by external forces and maintaining the structural integrity of the network. Compared to traditional crosslinked networks, this composite network, as the binder of Si anode, could significantly buffer the volume expansion of SNPs during charge-discharge cycles, thereby markedly improving the cycling stability of the Si anode.


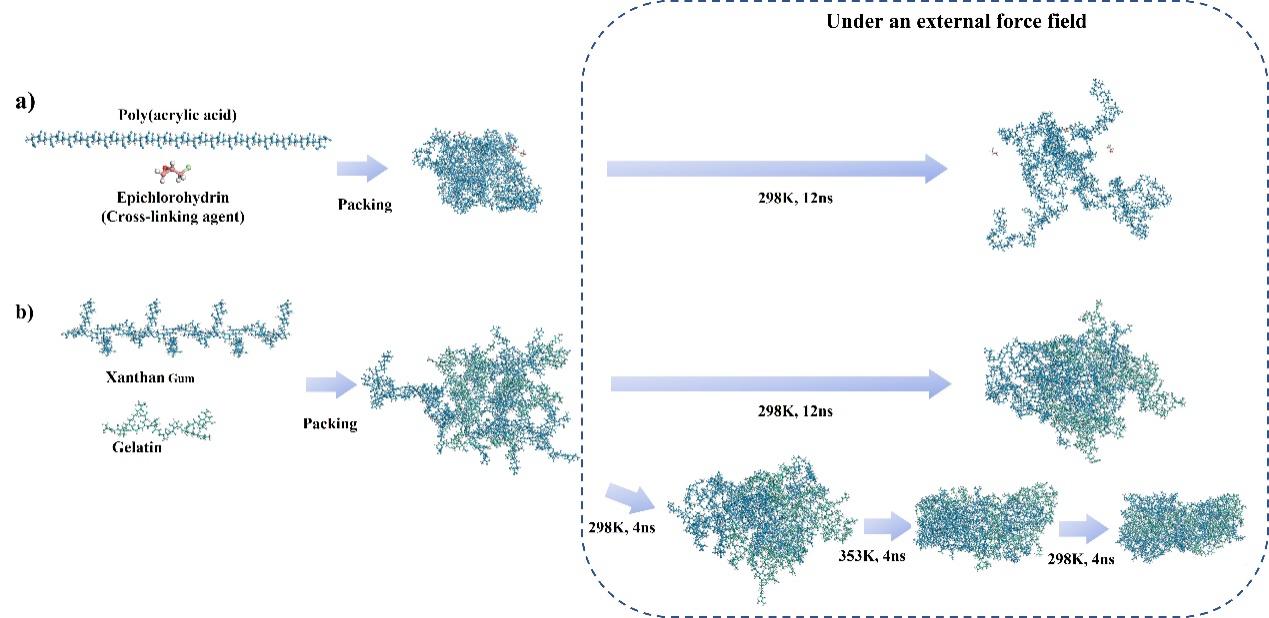


Figure S7. MD Simulations of different network structures under external force field and temperature conditions: (a) crosslinked PAA, (b) xanthan gum-gelatin


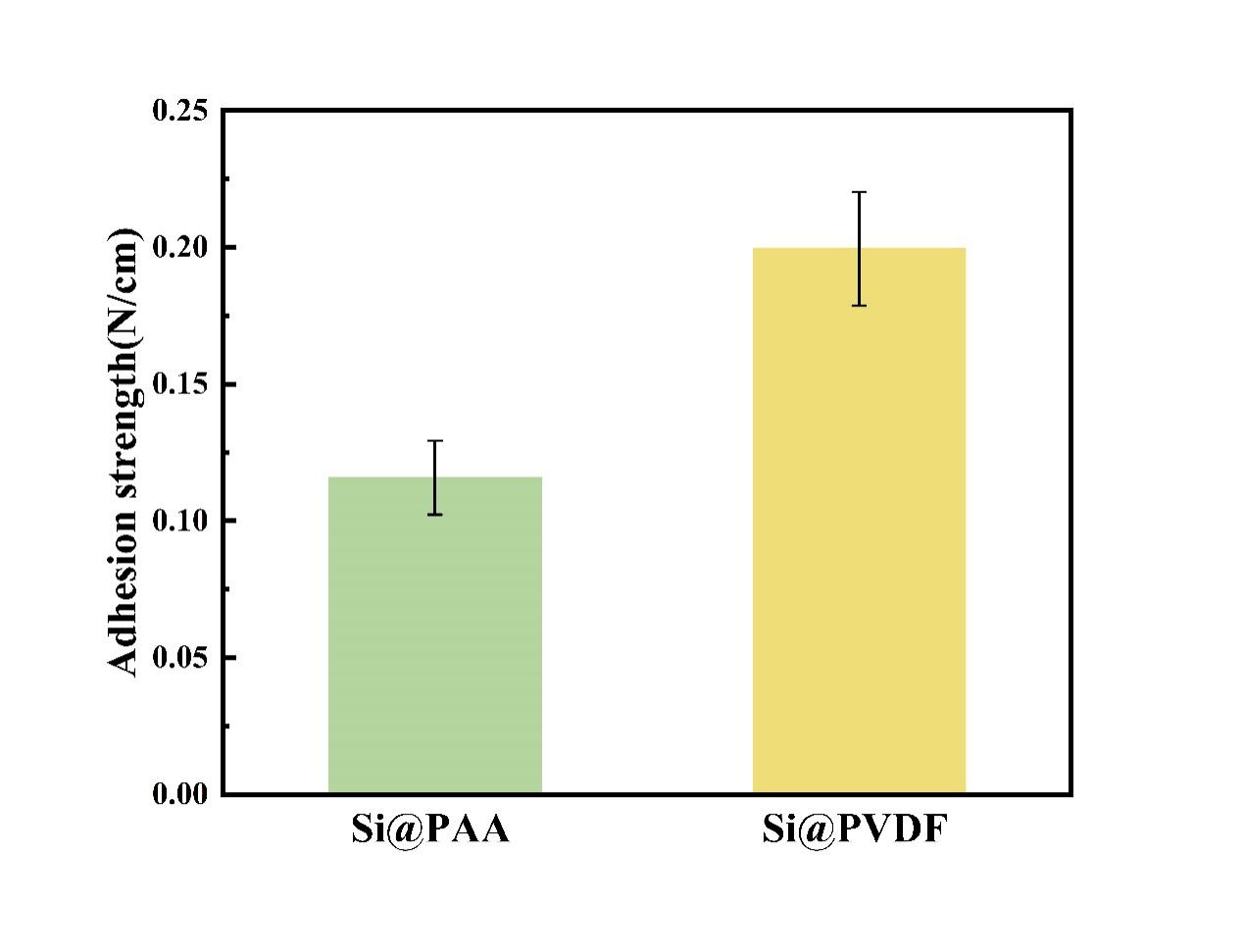


Figure S8. Adhesion of silicon anode foils prepared with PAA and PVDF as binders

After folding the Si@Xanthan Gum, Si@Gelatin, and Si@GX-AS samples twice, noticeable cracks were observed in the Si@Xanthan Gum and Si@Gelatin samples. In contrast, the Si@GX-AS electrode did not exhibit any cracks. This may be due to the salt immersion treatment increasing the hydrogen bonding and contact area between the binder and the copper foil interface, thereby enhancing the mechanical properties and electrode integrity of Si@GX-AS.


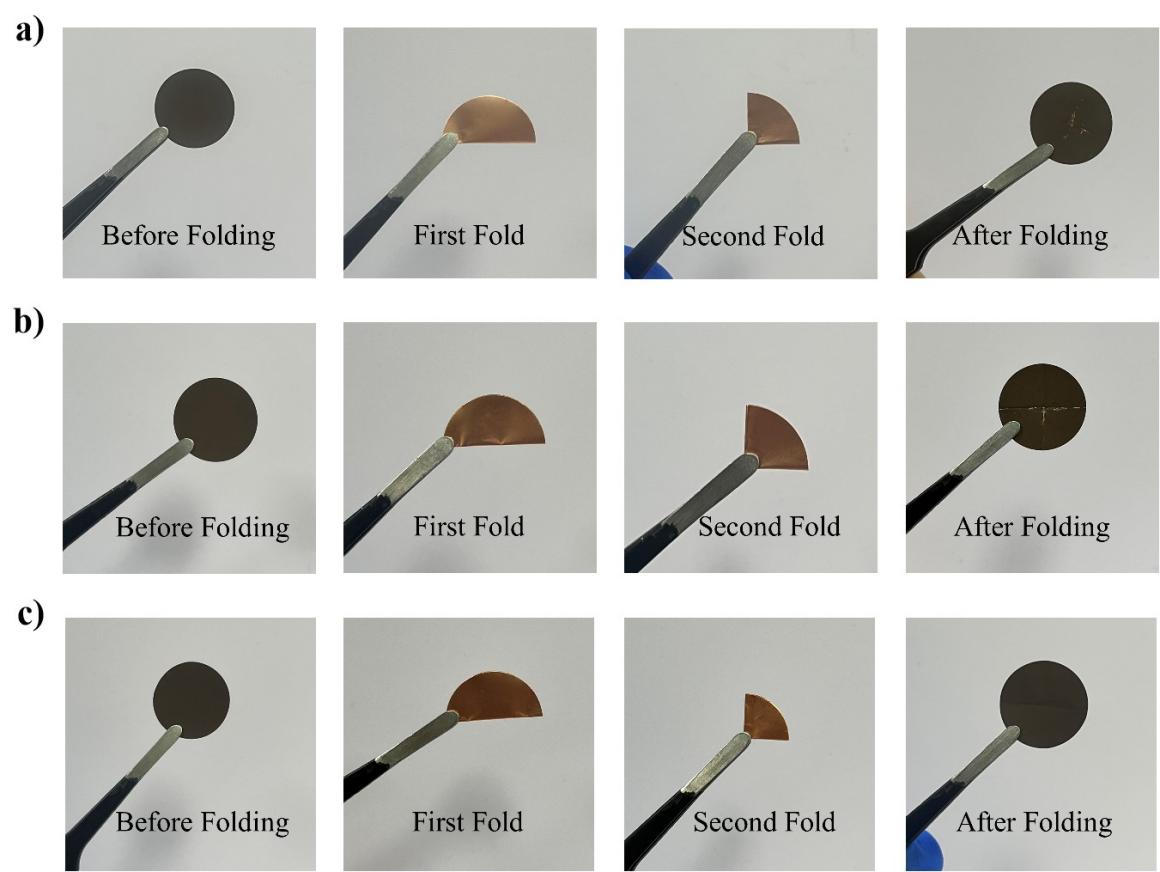


Figure S9. Photographs of Si@Xanthan Gum(a); Si@Gelatin(b); Si@GX-AS(c) anodes folded one time, two times and unfolded


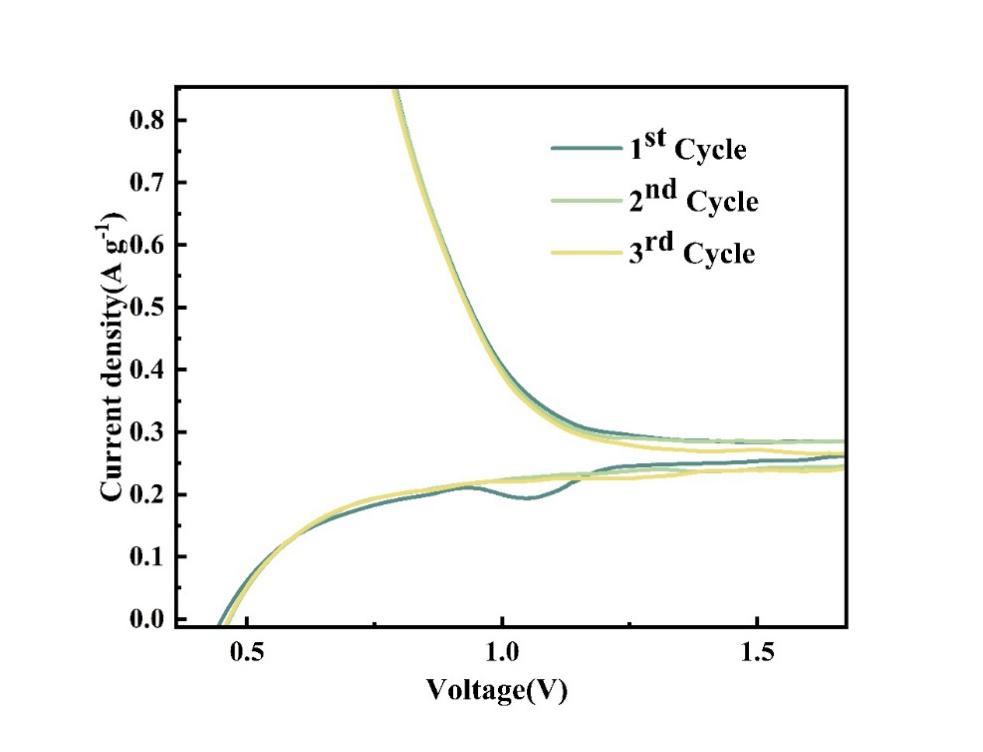


Figure S10. The magnified portion of CV curves of the Si@GX-AS anode at the scan rate of 0.1 mV s^-1^

An increase in the ratio of gelatin to xanthan gum from 2:1 to 5:1 has been observed to result in a notable enhancement in the cycle retention rate of the battery, rising from 0.75% to 44.60% after 300 cycles. This improvement is likely due to the optimal interactions between gelatin and xanthan gum at this ratio, which facilitate the formation of a uniform and stable network structure. This enhances the mechanical integrity of the binder, providing better structural support to the silicon anode and more effectively absorbing and releasing the stress and strain changes caused by the volume expansion of silicon. In conclusion, this significantly extends the cycle life of the silicon anode. However, when the ratio increases to 6:1, the excess gelatin may disrupt the uniform network structure. The additional gelatin molecules may not effectively integrate into the existing network, leading to an uneven distribution. This can result in weakened mechanical properties and reduced effective binding, causing the silicon particles to experience more significant volume changes during cycling. Consequently, this increases stress and potential cracking, ultimately reducing the cycle performance.


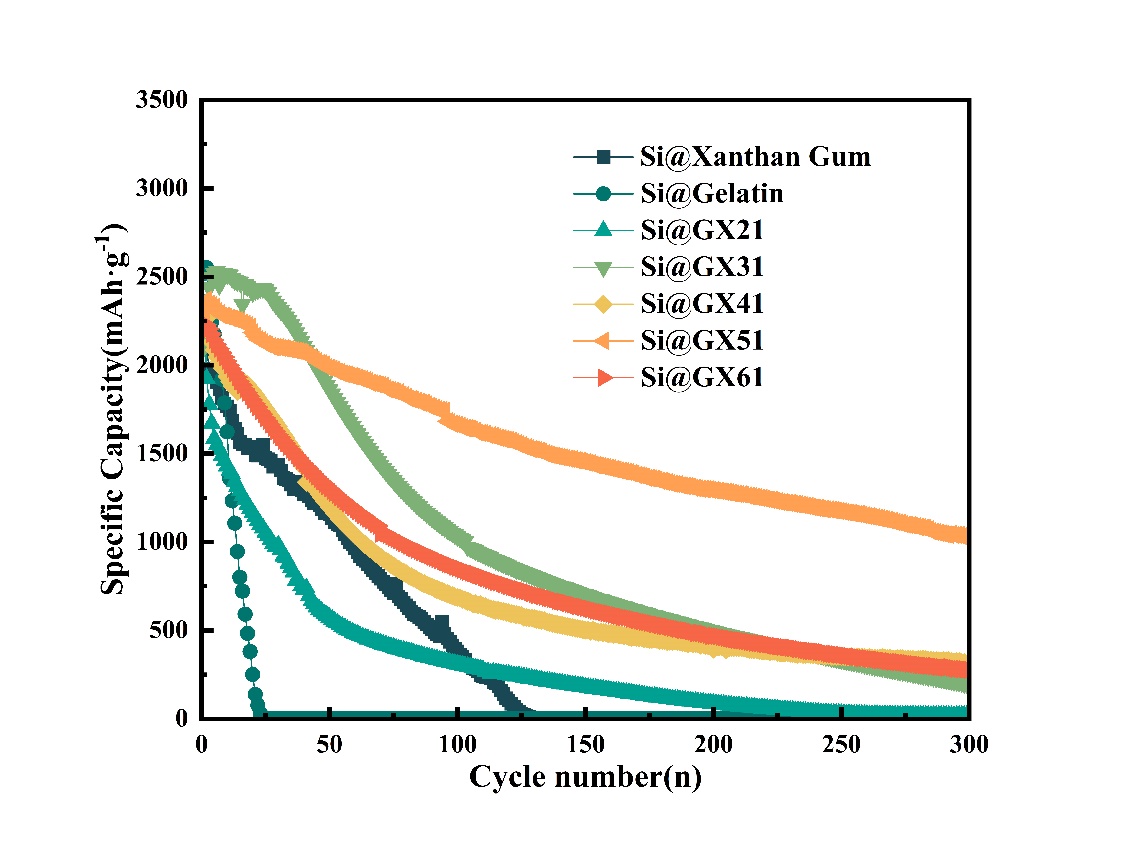


Figure S11. The cycling performance of silicon anode at 0.5 A g^-1^ for different binders

To evaluate the effects of different anions on the cycling performance of batteries, we immersed silicon-based anode sheets in ammonium chloride (NH_4_Cl), ammonium acetate (NH_4_Ac), and ammonium sulfate (AS) solutions for 12 hours. After washing off the residual salt solutions from the surface and drying, we assembled coin cells. The results showed that the stronger the salting-out effect, the better the capacity retention rate of the battery. According to the strength of the salting-out effect, the capacity retention rates were 80.65%, 61.01%, and 50.01%, respectively. This is because the binder materials soaked in solutions with a stronger salting-out effect formed a tighter network structure with stronger mechanical properties. A more stable network structure can better support the silicon anode, reducing the volume changes of silicon particles during charge-discharge cycles, thereby improving the cycling performance of the battery.


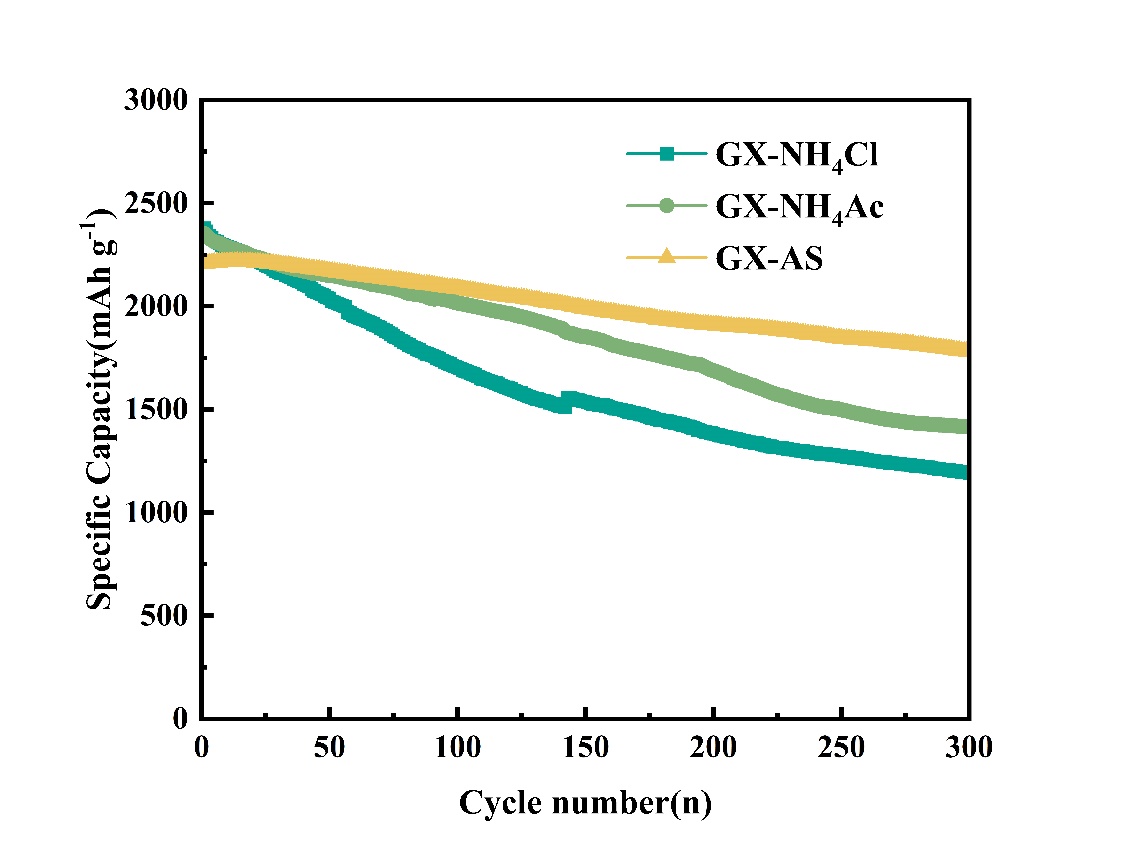


Figure S12. Cycle performance of Si@GX sheets after soaked in different Hofmeister salt solutions


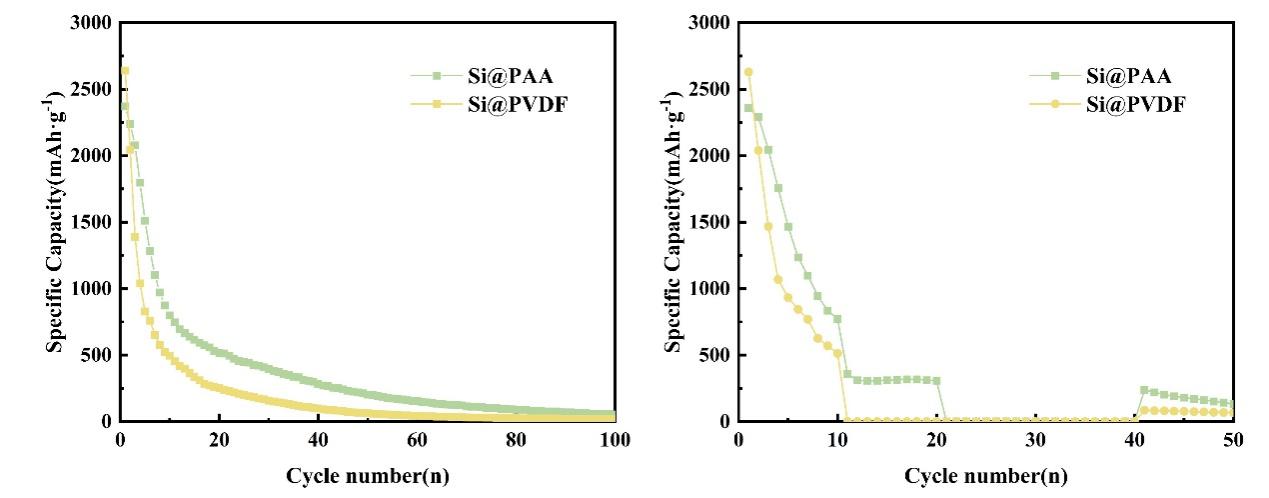


Figure S13. The cycling stability (a) and rate performance (b) of Si anode using PAA and PVDF as binders


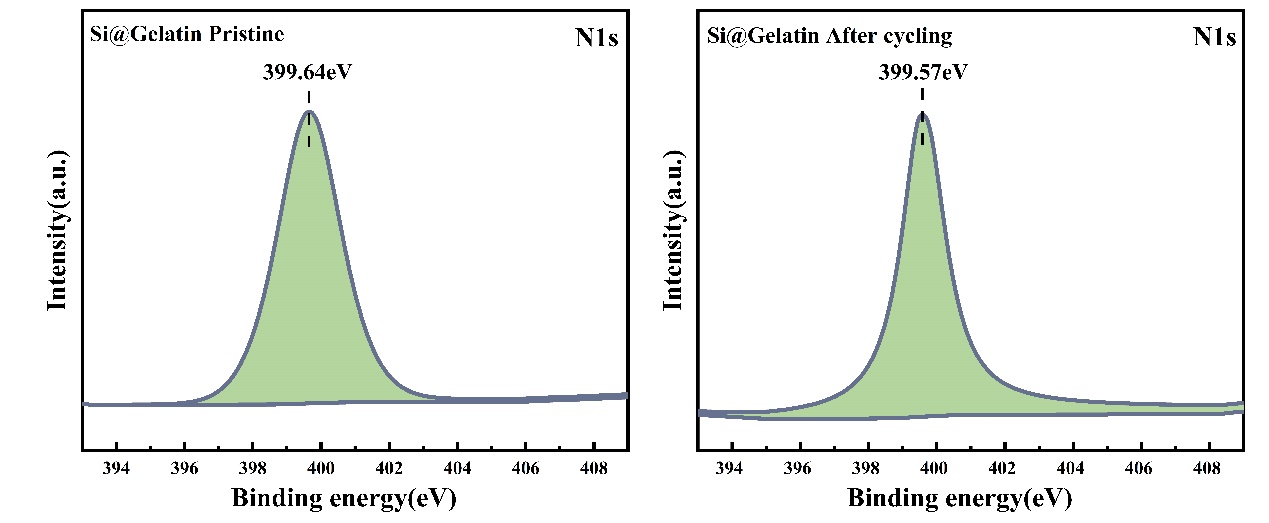


Figure S14. XPS spectra of N1s were obtain from Si@Gelatin before and after 100 cycles

Table S1. Comparison of electrochemical performance indices of the present work with other works

| Sample | Material  Characteristics | Number of cycle(n) | ICE(%) | capacity retention after cycles(%) | initial specific capacity(mAh g^-1^) |
| --- | --- | --- | --- | --- | --- |
| This work | Helical-entangled network | 300 | 92.7 | 88.4 | 2207 |
| Si@XG[5] | Rod-like polysaccharide | 200 | 77.2 | 72.2 | 2977 |
| Si@CS[6] | Linear polysaccharide | 100 | 91.7 | 83.6 | 2250 |
| Si@Alg-Ca-HBPEI[7] | Linear-branched dual network | 100 | 94.0 | 87.4 | 2750 |
| Si@GG-g-PAM[8] | Ion-conductive grafted polymeric network | 200 | 87.6 | 83.9 | 2341 |
| Si@CW-20[9] | Hydrogen-bonded flexible-rigid network | 100 | 89.5 | 82.5 | 3181 |
| Si@SA5H5[10] | One-pot synthesized flexible-rigid cross-linked network | 100 | 81.3 | 87.3 | 867 |
| Si@TUPN10[11] | Multifunctional interlocked network | 100 | 75.1 | 73.3 | 2088 |
| Si@GCA13[12] | Self-healing and reconfigurable structure | 200 | 93.0 | 62.0 | 3641 |
| Si@GE-PAA[13] | Gradient-distributed double-layer structure | 200 | 88.5 | 29.9 | 3345 |
| Si@PGC[14] | Dynamic cross-linking coordination conformation | 200 | 84.2 | 73.5 | 3606 |

Table S2. Corresponding experimental values by first-principles method for crystalline Li-Si alloys considered in this work

|  | Density  (g cm^-3^) | Young’s modulus  (GPa) | Poisson’s ratio | Volume expansion ratio |
| --- | --- | --- | --- | --- |
| Si | 2.236 | 152.2 | 0.21 | 1 |
| LiSi | 1.863 | 92.5 | 0.20 | 1.53 |
| Li_12_Si_7_ | 1.552 | 84.6 | 0.11 | 2.09 |
| Li_13_Si_4_ | 1.284 | 83.5 | 0.06 | 3.20 |
| Li_15_Si_4_ | 1.219 | 49.7 | 0.22 | 3.61 |
| Li_22_Si_5_ | 1.214 | 75.7 | 0.09 | 3.92 |

**Reference**

[1] E. Abraham, A. Nitzan, Physical Review Letters **2024**, 133 (26), 268001, <https://doi.org/10.1103/PhysRevLett.133.268001>.

[2] a) M. R. Tuchband, M. Shuai, K. A. Graber, D. Chen, C. Zhu, L. Radzihovsky, A. Klittnick, L. Foley, A. Scarbrough, J. H. Porada, M. Moran, J. Yelk, J. B. Hooper, X. Wei, D. Bedrov, C. Wang, E. Korblova, D. M. Walba, A. Hexemer, J. E. Maclennan, M. A. Glaser, N. A. Clark, Crystals **2024**, 14 (7), 583; b) R. Nigmatullin, A. del Campo, G. De Chiara, G. Morigi, M. B. Plenio, A. Retzker, Physical Review B **2016**, 93 (1), 014106, <https://doi.org/10.1103/PhysRevB.93.014106>.

[3] a) C. Zeng, P. Wu, J. Guo, N. Zhao, C. Ke, G. Liu, F. Zhou, W. Liu, Soft Matter **2022**, 18 (45), 8675, <https://doi.org/10.1039/D2SM01158A>; b) Y. Wu, Y. Mu, Y. Luo, C. Menon, Z. Zhou, P. K. Chu, S.-P. Feng, Advanced Functional Materials **2022**, 32 (15), 2110859, <https://doi.org/https://doi.org/10.1002/adfm.202110859>.

[4] B. Deng, J. Chen, S. Li, J. Liu, Z. Zhou, Z. Qin, H. Wang, M. Su, L. Li, Z. Bai, Int J Biol Macromol **2024**, 261 (Pt 2), 129706, <https://doi.org/10.1016/j.ijbiomac.2024.129706>.

[5] Y. K. Jeong, T.-W. Kwon, I. Lee, T. S. Kim, A. Coskun, J. W. Choi, Energy and Environmental Science **2015**, 8, 1224.

[6] P.-F. Cao, G. Yang, B. Li, Y. Zhang, S. Zhao, S. Zhang, A. Erwin, Z. Zhang, A. P. Sokolov, J. Nanda, T. Saito, ACS Energy Letters **2019**, 4 (5), 1171, <https://doi.org/10.1021/acsenergylett.9b00815>.

[7] J.-N. Wu, H.-X. Chen, C. Chen, H.-D. Li, H.-W. Zhang, B. Wang, Rare Metals **2023**, 42 (7), 2238, <https://doi.org/10.1007/s12598-023-02261-9>.

[8] Z. H. Li, G. Wu, Y. J. Yang, Z. W. Wan, X. M. Zeng, L. J. Yan, S. X. Wu, M. Ling, C. D. Liang, K. N. Hui, Z. Lin, Advanced Energy Materials **2022**, 12 (29), <https://doi.org/10.1002/aenm.202201197>.

[9] X. S. Sun, X. Y. Lin, Y. Wen, F. H. Dong, L. Z. Guo, Z. Q. Song, Z. T. Yang, H. Liu, X. Q. Li, X. Xu, H. X. Wang, Green Chemistry **2024**, 26 (18), 9874, <https://doi.org/10.1039/d4gc02666g>.

[10] B. Dong, H. Zhu, X. Cai, C. Guo, Y. Hao, L. Xi, Journal of Power Sources **2025**, 626, 235745, <https://doi.org/https://doi.org/10.1016/j.jpowsour.2024.235745>.

[11] J. H. Hwang, E. Kim, E. Y. Lim, W. Lee, J. O. Kim, I. Choi, Y. S. Kim, D. G. Kim, J. H. Lee, J. C. Lee, Adv Sci (Weinh) **2023**, 10 (30), e2302144, <https://doi.org/10.1002/advs.202302144>.

[12] Y. H. Tong, S. Y. Jin, H. Y. Xu, J. W. Li, Z. Kong, H. Jin, H. Xu, Advanced Science **2023**, 10 (2), <https://doi.org/10.1002/advs.202205443>.

[13] D. Zhang, Y. Ouyang, Y. Wang, L. Liu, H. Wang, J. Cui, M. Wang, N. Li, H. Zhao, S. Ding, Journal of Colloid and Interface Science **2024**, 673, 312, <https://doi.org/https://doi.org/10.1016/j.jcis.2024.06.086>.

[14] R. Ye, J. Liu, J. Tian, Y. Deng, X. Yang, Q. Chen, P. Zhang, J. Zhao, ACS Applied Materials & Interfaces **2024**, 16 (13), 16820, <https://doi.org/10.1021/acsami.4c00590>.
